# The Impact of a Nutritional Intervention on Glycemic Control and Cardiovascular Risk Markers in Type 2 Diabetes

**DOI:** 10.3390/nu16091378

**Published:** 2024-05-01

**Authors:** Tatiana Palotta Minari, Carolina Freitas Manzano, Lúcia Helena Bonalume Tácito, Louise Buonalumi Tácito Yugar, Luis Gustavo Sedenho-Prado, Tatiane de Azevedo Rubio, Antônio Carlos Pires, José Fernando Vilela-Martin, Luciana Neves Cosenso-Martin, Heitor Moreno, Juan Carlos Yugar-Toledo

**Affiliations:** 1 Department of Hypertension, State Faculty of Medicine of São José do Rio Preto (FAMERP), São José do Rio Preto 15090-000, SP, Brazil; 2 Department of Endocrinology, State Faculty of Medicine of São José do Rio Preto (FAMERP), São José do Rio Preto 15090-000, SP, Brazil; 3 School of Medical Sciences, State University of Campinas (UNICAMP), Campinas 13083-887, SP, Brazil; 4 Cardiovascular Pharmacology & Hypertension Laboratory, School of Medical Sciences, State University of Campinas (UNICAMP), Campinas 13083-887, SP, Brazil

**Keywords:** Type 2 diabetes mellitus, nutritional intervention, personalized nutrition, mediterranean diet, DASH diet, cardiovascular risk markers, blood pressure, total cholesterol, HDL cholesterol, LDL cholesterol, triglycerides, blood glucose, hemoglobin glycated, weight

## Abstract

Introduction: Nutritional management plays a crucial role in treating patients with type 2 diabetes (T2D), working to prevent and control the progression of chronic non-communicable diseases. Objectives: To evaluate the effects of individualized nutritional interventions on weight, body mass index (BMI), waist circumference (WC), waist-to-hip ratio (WHR), fasting blood glucose (FBG), hemoglobin A1c (HbA1c), total cholesterol (TC), LDL cholesterol (LDL-C), HDL cholesterol (HDL-C), triglycerides (TGs), systolic blood pressure (SBP), diastolic blood pressure (DBP), and heart rate (HR)} over 12 months and subsequently at follow-up (15 months). Methods: This longitudinal experimental study (without randomization and blinding) enrolled 84 sedentary participants with T2D (both sexes, aged 18–80 years). They were divided into a control group of 40 participants who received only medical consultations, and an intervention group of 44 participants who received the same medical care along with a nutritional assessment. Consultations occurred quarterly from August 2020 to November 2022 (first–twelfth month), with six to nine patients per session. Subsequently, a follow-up was conducted from December 2022 to November 2023, during which the intervention group had only medical care (during the 12th–15th months). Personalized dietary planning was inspired by the Mediterranean/DASH diets adapted to Brazilian foods and socioeconomic cultures. Statistical Analysis: Normal variables were compared between groups for each time point and also within each group across different time points using a two-way ANOVA (repeated measures for intragroup) followed by the Šídák post hoc test. Non-normal variables were compared between groups for each time point using Kruskal–Wallis followed by the Dunn post hoc test, and within each group across different time points using Friedman followed by the Dunn post hoc test. Data with a Gaussian distribution were presented as mean ± standard deviation (SD), and data with a non-Gaussian distribution were presented as median ± interquartile range (IQR). For all cases, α < 0.05 and *p* < 0.05 were adopted. Results: In the intervention group, significant reductions were observed between the first and twelfth month for all parameters (*p* < 0.05), (except for TC), along with an increase in HDL-C (*p* = 0.0105). Conversely, in the control group, there was a significant increase in HbA1c, weight, BMI, FBG, and WHR (*p* < 0.05) between the first and twelfth months. Regarding the comparison between groups, there was a significant difference for all analyzed parameters (*p* < 0.05) from the first to the twelfth month. In the follow-up, differences were also observed (*p* < 0.05), except for BMI (*p* > 0.05). Conclusion: The individualized nutritional intervention improved eating habits, anthropometric, biochemical, and cardiovascular markers in T2D over 12 months, with sustained results during follow-up. The dietary plan inspired by the Mediterranean and DASH diets demonstrated good adaptation to the Brazilian food culture and the patients’ socioeconomic contexts. Consistent monitoring and personalized nutritional management are essential for optimizing long-term outcomes. However, more clinical trials are necessary in order to optimize the level of evidence for longitudinal interventions.

## 1. Introduction

### 1.1. Epidemiology

Type 2 diabetes mellitus (T2D) is a highly prevalent chronic disease worldwide and represents one of the biggest public health problems of the 21st century [1]. Its high incidence and prevalence rates are mainly attributed to the population’s aging and inadequate lifestyles [2,3]. In 2021, approximately 537 million individuals (between 20 to 79 years old) were diagnosed with the disease and the probability is that these data will increase to 783 million by 2045. In Central and South America, it is estimated that there are 32 million patients with diabetes, with 15.8 million just in Brazil, with projections of this increasing to 49 million by 2045 [1].

Simultaneously, there is a worsening of this scenario with the progressive weight increase of the population [2,3]. According to the WHO, by 2025, it is estimated that 2.3 billion adults around the world will be overweight, with 700 million individuals being with obesity [4]. Obesity is a multifactorial, complex, and recurrent disease, predisposing the individual to the development of non-communicable chronic diseases (NCDs), such as T2D, hypertension, and cardiovascular diseases [2,3,4,5,6]. According to the research from 2023, approximately 61.4% of the Brazilian population was overweight and 24.3% were obese [7]. An article published in February 2024 pointed out that approximately 1 billion people were diagnosed with obesity in 2022 [6]. In light of these growing numbers and this worrying scenario, it is up to society to develop strategies and public policies for the prevention, control, and treatment of NCDs [2,6,7,8,9].

### 1.2. Nutritional Management

Among the therapeutic tools available for controlling glycemia, blood pressure, and cardiovascular parameters, diet, physical exercise, and pharmacotherapy stand out [10,11,12]. Weight loss is one of the main points of congruence highlighted in all types of management [13], especially for patients with T2D who are overweight or are affected by obesity [2]. This premise is based on short-term studies, which point out various benefits of fat loss promoted by caloric restriction in the body [13,14,15,16,17,18,19]. A negative energy balance is capable of promoting various anti-inflammatory effects [20], such as (1) reducing cytokine production; (2) inactivating Toll-Like Receptor 4 (TLR4); (3) increasing lipolysis, beta-oxidation, and the production of ketone bodies; (4) reducing leptin resistance; (5) improving microbiota modulation (an increase in short-chain fatty acids, a reduction in Lipopolysaccharide (LPS) and Trimethylamine N-Oxide (TMAO); and (6) stimulating the production of brown adipose tissue. However, the big question would be the best strategy to promote quality caloric restriction (without leading the patient to malnutrition) and how to sustain new healthy habits in the long term [19,20,21,22].

### 1.3. Personalization/Nutritional Precision

The evidence suggests that there is no ideal nutritional strategy or percentage of calories, carbohydrates, proteins, fats, and fibers for the management of T2D [10]. The type of conduct should include (1) an individualized dietary prescription adapted to the patient’s eating habits, preferences, objectives, culture, religion, and economic situation [2,10]; (2) a nutritional re-education based on the importance of the balance of concentrations of fats, carbohydrates, and proteins to control the disease [23,24,25]; (3) the flexibility of the food repertoire based on progress or therapeutic success aiming at adherence to and sustaining results in the long term [10]; (4) the engagement and self-monitoring of the patient in relation to their glycemic rates, aiming to improve their meal times, carbohydrate counting [24], food choices, and adherence to the intervention [14]; (5) care with the language and communication used, aiming to mitigate stigmas and prejudices, thus optimizing the reception of the approach by patient, especially for those who are also affected by being overweight and having obesity [2,13,14,15,16,17,26]; and (6) interdisciplinary work [2,3], with encouragement regarding the regular practice of physical exercises [2,17] and the correct use of medications [14,16].

### 1.4. Dietary Patterns

Even without an ideal intervention, several references unanimously affirm that the Mediterranean and DASH (Dietary Approaches to Stop Hypertension) diets can bring promising results in improving patients’ quality of life, reducing the mortality rates of patients with NCDs [10]. Epidemiological research also correlates these two dietary patterns with lower incidences of T2D, greater weight loss, the improvement of biochemical parameters, and sustaining these results in the long term [27,28,29,30,31].

### 1.5. Mediterranean Diet

The Mediterranean diet was created around 1960 in the region of Greece and southern Italy [32], currently comprising one of the highest levels of scientific evidence available in the literature [10]. It is composed of in natura and minimally processed foods, with moderate carbohydrate contents, a moderate fat intake (a reduction of saturated fats to be <7% of the total energy value and an increase of poly/monounsaturated fats), and high amounts of fibers. The food repertoire includes fruits, vegetables, legumes, greens, tubers, grains, whole cereals, oilseeds, seeds, fish (as a primary protein source), skinless poultry, skimmed dairy products, vegetable oils (olive oil as a primary fat source), spices, aromatic herbs, teas, wine in moderation (a maximum of one glass for women and two glasses for men/day), and others [10,33]. Numerous studies have reported the protective effect of this diet on metabolic disorders, chronic diseases, and mental health [2,3]. The key point would be its good adherence to the regimen, wide food variability, good palatability, and its improvement/maintenance of general health parameters in the long term [10]. Other interesting findings are related to the reduction in fasting glycemia, HbA1c (hemoglobin glycated), LDL-C (Low-Density Lipoprotein Cholesterol), TC (total cholesterol), and weight. Studies also correlates this dietary pattern with a reduction in the predisposition for NCDs, the incidence of T2DM, cardiovascular risk, and the use of medications, along with an increase in life expectancy [12,34,35,36].

### 1.6. DASH Diet (Dietary Approaches to Stop Hypertension)

The DASH diet is a nutritional intervention that was postulated around 1990 by the National Institute of Health (NIH), initially targeting body weight reduction, so that there is a substantial attenuation of blood pressure [37]. This dietary pattern has characteristics similar to the Mediterranean diet, but alcohol consumption is not widely encouraged and sodium restriction tends to be a little bit higher depending on the type of associated cardiovascular and/or renal disease (2300 to 1500 mg/day) [10,12]. In addition, the DASH diet advocates the intake of larger portions of vegetables, fruits, and whole grains [10,38] and can also reduce blood pressure levels, weight, TC, LDL-C, and HbA1c in T2D [39,40,41,42,43]. These benefits are closely linked to its high fiber, low saturated fat, and moderate carbohydrate contents. It also proves to be effective in sustaining long-term results through the wide variability of food groups and good adherence to the regimen [43,44,45,46].

### 1.7. Sustainable and Economically Viable Strategies

The great challenge of T2D management in public health is to reconcile simple and economically viable strategies in patients with high socioeconomic vulnerability [23,25]. It is the role of the nutritionist to develop practical meal plans compatible with the patient’s reality, educating them to understand the nutritional composition, labeling, and identification of foods, aiming for total autonomy and more assertive choices [10]. In this way, the patient becomes able to understand that healthy eating does not have to be expensive and that in most cases, ultra-processed foods (cakes, sweets, biscuits, soft drinks, snacks, nectar, and others) have triple the calories and prices compared to in natura and minimally processed foods (vegetables, fruits, cereals, grains, legumes, tubers, roots, lean protein sources, oilseeds, spices, aromatic herbs, and others) [2,25]. It is noted that dietary interventions do not need to have little food and make the patient go hungry, it is possible to prescribe meal plans that combine taste and health, within contexts that involve socioeconomic vulnerabilities [25].

Lifestyle changes can involve various simple strategies that do not require public spending and/or major logistical complexities [23,25]. There are several factors that can impact postprandial blood glucose that should be considered when treating a patient with T2D, such as (1) the individual’s glycemic response, which is highly correlated with genetics and microbiota [47]; (2) the method of preparation and the cooking time of carbohydrate-source foods [24]; (3) the addition of other types of foods to the meal, such as vegetables, legumes, proteins, poly- and monounsaturated fats, and even water (or other liquids) [10,23,24]; (4) the order of the ingestion of these foods [48,49]; (5) the daytime feeding windows [50]; (6) stress management and sleep deprivation [51]; (7) the encouragement to practice postprandial physical exercises [52,53]; and (8) the mindful eating and chewing of food [54,55,56].

The simple fact of paying attention to the order of food intake is an excellent economically accessible option for daily life [10]. Research points out that eating vegetables and protein-source foods at the beginning of meals and later ingesting carbohydrate sources can significantly affect postprandial glucose and insulin peaks [47] in prediabetes [48] and T2D [49]. The potential effect for this would be related to the important role of fiber in attenuating and significantly impacting this process. However, studies caution generalizing the results, as some patients may not obey the results of the population average [48,49].

Another interesting topic that has been highlighted in the literature is the impact of meal timing on glycemic control, which is that, apparently, having a daytime feeding window (7 a.m.–7 p.m.) has been shown to be potentially effective in improving daily glycemic response and insulin resistance, compared to late feeding windows (12 p.m.–12 a.m.) [50]. Studies point out that skipping breakfast can be associated with being overweight, having obesity, a persistent increase in arterial stiffness, and a greater likelihood of compensating with food intake in later meals, thus decreasing the quality of eating and increasing caloric consumption for the rest of the day [10,50]. In this same line of reasoning, research shows that sleep changes have also become lifestyle risk factors, especially for patients with T2D. Over the course of the 21st century, it has been noticed that approximately 20% of individuals do more night work, 33% do not sleep more than 6 h per night, have recurrent insomnia/or night awakenings, and 69% have a social jetlag experience. Epidemiological studies have shown that circadian disruption increases the risk of NCDs, a higher weight, cardiovascular risk, and glycemic dyscontrol. Therefore, going to bed early is also another simple strategy that can optimize glycemic control in T2D [51].

Other studies have demonstrated the potential effect of walks after meals, aiming to optimize glycemic control within the time range of the appropriate target [52]. The explanation would be that exercise contributes to peripheral glucose uptake postprandially, through the increase of adrenaline and the release of calcium in the sarcoplasmic reticulum, which collaborate to activate the Adenosine Monophosphate-Activated Protein Kinase (AMPK) pathway and optimize the functioning of glucose transporters. The most consistent benefits were observed in aerobic exercises (45 min) with moderate intensities and also in resistance training [52]. In this sense, it is suggested to increase energy expenditure after meals with the highest energy content of the day (especially carbohydrates), aiming to attenuate glycemic responses [53].

In addition to the development of a meal plan, other subjective practices have been gaining prominence in the literature to mitigate dietary intake and indirectly impact weight loss [10]. This is the case with mindful eating, which consists of (1) having full attention during meals; (2) observing food in a gentle and non-judgmental way; (3) respecting hunger and satiety signals; (4) chewing, appreciating the textures and the flavors of food; (5) turning off the TV, cell phone, and radio during meals; (6) worrying about the origin and quality of food; (7) recognizing the importance of taking care of your body; and (8) eating in company [55,56]. The patient can also learn to identify the causes of food intake, applying the use of a food diary [23].

### 1.8. Literature Gap

Despite the good reputation of the Mediterranean and DASH diets in the literature, there are still controversies, both in relation to their adaptation to other food cultures worldwide, and to their socioeconomic adequacy, especially in developing countries [10,23,39]. In addition, there are few studies addressing the theme of “personalization/precision dietetics”, which overthrow the use of generalist nutritional protocols [47]. There is also a certain difficulty in following patients in the long term, due to the various methodological limitations faced by these types of longitudinal studies, especially those whose main objective is to promote negative energy balance (examples include dropouts, deaths, the loss of engagement, demotivation, the loss of palatability, physiological/hormonal regulation after weight loss, increased appetite, and other reasons) [17,18,19,20,21,22].

Furthermore, although emerging and economically viable strategies present good results, there are still few studies that point out large effects and robust sample representativeness, especially in the long term [47,48,49,50,51,52,53,54,55,56]. In this sense, the main motivation for the execution of the project came from the premise of carrying out a dietary intervention that would significantly impact the lives of patients of the Unified Health System (also called the SUS) of the Outpatient Clinic of the Base Hospital (FUNFARME), in São José do Rio Preto, SP/Brazil. This would provide an individualized, humanized, and sustainable nutritional management in the context of public health. Secondarily, it is extremely important for the nutritionist to understand and adapt to the reality of the patients whose social representation mirrors a large part of the Brazilian population, thus enabling economically effective and accessible strategies (both for patients and for reducing public spending on the SUS). It is important to see and take care of the individual as a whole, that is, to understand not only what happens in physiological and metabolic reactions, but also what is in the patient’s behavioral and emotional plan. Therefore, it is extremely important that nutritional management is inserted into lifestyle change programs, especially in the context of public health, aiming to optimize and sustain results in the long term.

## 2. Objectives

The overall objective was to evaluate the effects of individualized nutritional interventions, inspired by the Mediterranean/DASH diets and adapted to the Brazilian food culture, on the anthropometric, biochemical, cardiovascular parameters of participants with T2D in the long term.

### Specific Objectives

There was a specific objective to evaluate the changes in anthropometric (weight, BMI, waist/hip ratio, and waist circumference), biochemical (fasting blood glucose, glycated hemoglobin, total cholesterol, LDL cholesterol, HDL cholesterol, and triglycerides), and cardiovascular (systolic blood pressure, diastolic blood pressure, and heart rate) data of participants with T2D submitted to a nutritional intervention over 12 months, and sequentially, to verify the stability of the results post-intervention in the 15th month (follow-up).

## 3. Materials and Methods

### 3.1. Case Study/Participants

**Figure 1:**
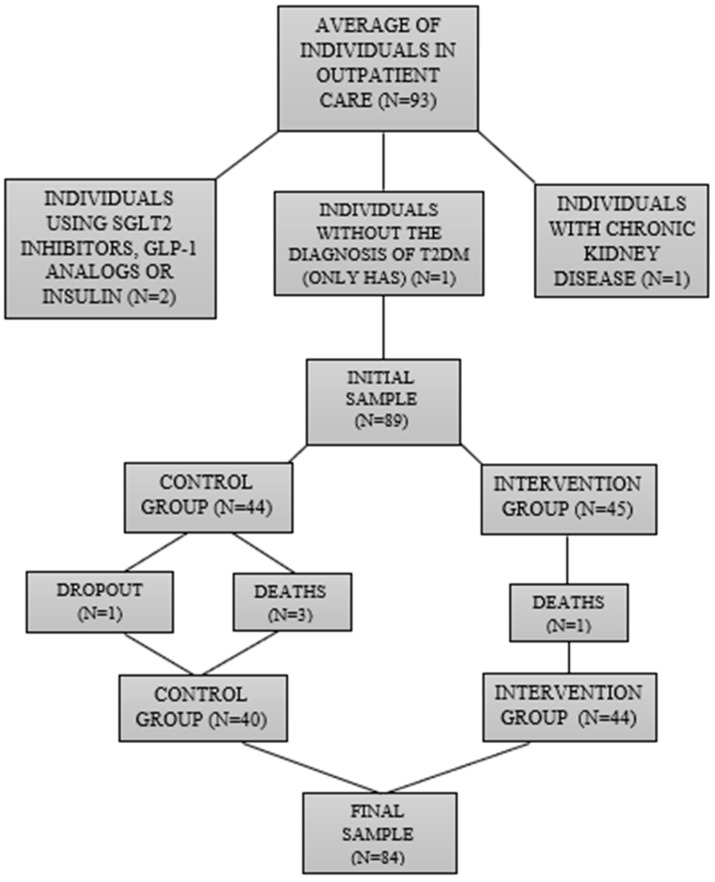
Flowchart of the Research participants sample selection.

Participants diagnosed with T2D were recruited from the Hypertension Clinic and the Endocrinology Clinic, both linked to the Base Hospital (FUNFARME) of São José do Rio Preto, SP/Brazil. The sample size was based on the concept of a “convenience sample”, which means the use of available participants interested in participating in the research at a certain place and time. In this regard, initially, the proposal was to collect as many patients as possible of both sexes. Therefore, the total number of patients who were being monitored in both clinics at the start of the study was 93 individuals. After going through the selection criteria, the sample size was reduced to 89 participants, who were divided into two groups: (1) Control: 44 participants who underwent only conventional medical evaluations; (2) Intervention: 45 participants who received the same medical care, concurrent with nutritional evaluations. Due to complications (deaths and dropouts throughout the project), the number of individuals in the control group was reduced to 40 and in the intervention group to 44. Thus, the final sample size totaled 84 participants, which means 40 from the control group and 44 from the intervention group. Figure 1, below, represents the complete flowchart of the research sample selection.

#### 3.1.1. The Inclusion Criteria for Participants Were as Follows

- Age: Between 18 and 80 years old.
- Diagnosis: Confirmed to have type 2 diabetes (T2D), indicated by fasting blood glucose levels ≥126 mg/dL and a glycated hemoglobin ≥ 6.5%.
- Gender: Both male and female participants are included, aiming to expand the sample number and collect as many participants as possible.
- Commitment: An availability to participate in quarterly meetings for a duration of 36 months.
- Nutritional Status: A Body Mass Index (BMI) greater than 24.9 kg/m^2^.
- Lifestyle: Sedentary.

#### 3.1.2. The Exclusion Criteria for Participants Were as Follows

- Individuals who had difficulties answering the requested instruments.
- Those who demonstrated impediments to regular data collection.
- Individuals without a confirmed diagnosis of type 2 diabetes (T2D).
- Those who were using insulin therapy.
- Individuals who were using Sodium-Glucose Transport Protein (SGLT-2) inhibitors and/or Glucagon-like peptide-1 (GLP-1) analogs.
- Those diagnosed with Chronic Kidney Disease (CKD).
- Individuals who were eutrophic or malnourished.
- Those who practiced physical exercise for more than 150 min during the week.

### 3.2. Materials and Procedures

#### 3.2.1. Study Design and General Information

This prospective and longitudinal study was comprised of experimental work that did not involve randomization or blinding. The patients in both the control and intervention groups were part of the Hypertension and Endocrinology Outpatient Clinic at the Base Hospital (FUNFARME) of São José do Rio Preto. The first contact with the participants occurred in the outpatient consultation room, where the most detailed information about the research project (the dates, duration, procedures, and other variables) was provided. After understanding and agreeing to participate in the intervention, everyone signed the Informed Consent Form (ICF). The detailing of the ethical aspects is described in the topic “3.3.11. Ethical Aspects”.

Nutritional consultations took place quarterly over 3 years (2020 to 2023). The outpatient services were traditionally held weekly, but due to the COVID-19 pandemic, they were adjusted to biweekly consultations, on Wednesdays, from 8 a.m. to 12 p.m., from 2020 to 2023, totaling 12 months of data collection and 3 months of post-intervention follow-up (a total of 15 months). On each Wednesday, an average of 6 to 9 patients were seen, depending on the availability of the schedule and outpatient flow. The average duration of each meeting was approximately 1 h per participant. In addition, individuals were also reminded of the consultations by phone by the secretaries of the General Outpatient Clinic of the Base Hospital-FUNFARME. A communication link was established between the researcher and the participants via phone and/or text messages or mobile video calls, aiming to clarify doubts if necessary. Data collection began after the first lockdown of the COVID-19 pandemic, on 16 August 2020, so there was no compromise in the measurement of anthropometric data, blood pressure, and biochemical tests, as face-to-face services returned to usual operation in August. In the second lockdown (during March and April 2021) there was also no interruption of services, as the researchers had written permission to travel through the streets and the Outpatient Clinic. During the pandemic, all criteria for social distancing, mask use, and personal hygiene were respected, aiming at the health integrity of patients and researchers.

In the first year of service (between 2020 and 2021), the nutritional consultations were detailed and intensive, with the aim of optimizing the regularization of the food plan, transmitting knowledge, and making necessary adjustments. In all quarterly consultations, anthropometric information, biochemical parameters, cardiovascular parameters, participants’ food diary, and the application of research protocols were collected. Other characteristics of the population samples such as patients’ race, socioeconomic level, level of physical activity, details of their diet quality, the presence or absence of COVID-19 infection, sleep duration, nocturnal awakenings, and the habit of having breakfast were also collected. In some cases, it was necessary to adjust the food plan, due to eventual intercurrences, preferences, and flexibility, aiming to optimize long-term adherence. All information was obtained through consultations, medical records, and, in the last cases, via video call.

In the year 2022, the consultations had a more educational and guiding nature, aiming to follow the patient’s evolution and recall the concepts previously transmitted. Finally, in 2023, the researcher avoided giving guidance to the participants, as it was the follow-up period, thus minimizing possible interferences in the results. The data collection ended on 15 November 2023. Therefore, given the constraint of attending to patients in biweekly appointments, it was necessary to carry out an intervention over 3 years to complete a data analysis spreadsheet within 15 months (12 months of intervention + 3 months of follow-up).

#### 3.2.2. Nutritional Interventions

The quarterly meetings and nutritional interventions were based on nutritional evaluations (anthropometric, biochemical, clinical, and dietary analyses) and quantitative and qualitative nutritional strategies, through the elaboration of a personalized food plan, based on the guidelines postulated in the following references: Dietary Guidelines for the Brazilian Population—Ministry of Health/Brazil [25]; International guidelines from the American Diabetes Association 2023 [2,11,12,13,14,15,16,17], the European Society of Cardiology 2023 [3], and the International Diabetes Federation 2021 [1]; the National guidelines from the Brazilian Diabetes Society (SBD) 2023 [23] and the Brazilian Society of Cardiology 2021 [12]; the Mediterranean Diet [28,29,30,31,32,33,34,35,57,58,59,60]; the Diet Approach to Stop Hypertension-DASH [37,38,39,40,61,62,63]; the Lifestyle Interventions in T2D-“Look AHEAD (Action for Health in Diabetes)” [18,19,20,21]; the Carbohydrate Counting Manual-SBD 2021 [24].

Below is Table 1, which contains the summary of the meetings of this project

#### 3.2.3. Detailing of Protocols and Procedures

(1) Nutritional Anamnesis Protocol: Analyzes the clinical and dietary parameters, which assist in the careful evaluation of the participant, and can even alert for some risk signs and diseases that are related to their diet [64].
(2) Protocol for anthropometric, biochemical, and clinical evaluation: Analyzes the anthropometric, biochemical, clinical, and other general parameters’ information that assists in the complete evaluation of the participant [65].
(3) Dietary evaluation protocol (habitual food recall): Analyzes the patient’s usual food intake, allowing for the careful evaluation of both quantitative and qualitative aspects and secondarily calculations, estimates, possible deficiencies, and excesses from the patient [64].
(4) Sociodemographic research protocol: Provides general data, such as age, sex, socioeconomic level, race, profession, and other details. To designate social classes, the following classifications were considered: Class A (those who earn more than 20 minimum wages); Class B (from 10 to 20 minimum wages); Class C (from 4 to 10 minimum wages); Class D (from 2 to 4 minimum wages); and Class E (receives up to 2 minimum wages) [66]. In 2020 the value of a minimum wage was BRL 1039. Note: BRL 1039 are equivalent to approximately USD 207 or EUR 191, considering the values of currencies that were quoted at the end of 2020.
(5) Nutritional guidelines for participants with T2D (APPENDIX 1 in Supplementary Materials): Offers guidelines, tips, and general clarifications for disease control directed at participants with T2D.
(6) An individualized food plan was prepared and delivered at the time of consultation (APPENDIX 2 in Supplementary Materials).

##### Qualitative Aspects:

The dietary pattern was inspired by the Mediterranean and DASH diets, which include: fruits/vegetables/legumes (various types according to the harvest period); roots/tubers (various pumpkins-kabocha/squash, potatoes, cassava, sweet potatoes, carrots, and beets); cereals (mainly rice, couscous, tapioca, and French bread); legumes (carioca and black beans); grains/seeds/oilseeds (pumpkin seeds, sunflower, and peanuts); lean proteins (various types of fish, chicken breast, pork loin/fillet, and occasionally lean beef); eggs; skimmed dairy products (skimmed milk and yogurt, fresh cheese, and ricotta); textured soy protein (vegetarian option); vegetable oils (canola, sunflower, and soy); aromatic herbs/spices; tea; coffee; and water [10,57,58,59,60,61,62,63]. The foods included in the food plan were also standardized to the Brazilian food culture, socioeconomic level, and the individuality of the SUS participant, also taking into account the cost–benefit, which means foods that are in the harvest period and economically accessible [25]. Although no participant has officially declared a preference for a vegetarian/vegan dietary pattern, options for plant-based protein sources were also offered, aiming to maximize the repertoire of options and reduce costs.

##### Energy Calculations and Estimate Formulas:

(a) The basal metabolic rate (BMR) of patients was calculated using the Mifflin-St Jeor Formula [67] since the groups were largely composed of individuals with an obesity nutritional status: BMR (men) = (10 × weight in kg) + (6.25 × height in cm) − (5 × age in years) + 5; BMR (women) = (10 × weight in kg) + (6.25 × height in cm) − (5 × age in years) − 161.
(b) The adjusted weight (AW) formula was also used for patients with obesity AW (kg) = [current weight (kg)-ideal weight (kg)] × 0.25 + ideal weight (kg) [68].
(c) The total energy expenditure (TEE) was calculated using the formula TEE = BMR × PAL. The PAL (physical activity level) was considered to be 1.2, since all selected patients were sedentary [69].
(d) The total energy value (TEV) of the baseline was calculated from the estimate of the total daily caloric value collected in the habitual food recall of the participants. For the calculation of the TEV of the new food plan, a deficit of 500 kcal was estimated in relation to the TEV baseline, remembering that all calculations were adjusted to the individuality and objectives of each patient. All values (BMR, TEE, and TEV) were expressed in calories (Kcal).

##### Dietary Prescription

(a) Carbohydrates: 40–50% of the TEV or 1.5–2.5 g/kg/weight of carbohydrates or up to 130 g of carbohydrates per day (Note: 40% for decompensated patients; 45–50% for controlled patients or those who needed flexibility in the food plan) [2,3,10,23];
(b) Proteins: 15–25% of the TEV or 1.4–2.0 g/kg/weight of proteins [2,3,10,23];
(c) Fats: 25–35% of the TEV {<7% saturated (or 5 to 6% associated with severe heart diseases), 10% polyunsaturated, 10–20% monounsaturated} or 0.5–1.5 g/kg/weight of [2,3,10,23];
(d) Fibers: minimum of 14 g/1000 kcal [2,3,10,23];
(e) Sodium: <2300 mg of sodium or 1500 mg/day (marked restriction for heart diseases, heart failure, and other indications) [2,3,10,23].
(f) Supplementary documents: a food diary [70] and “10 Steps to Healthy Eating” [25]. A food diary is used by nutritionists to maximize the participant’s self-knowledge in relation to their eating habits, involving an awareness of the quality, quantity, and frequency of food intake. The participant was instructed to note all the times of their meals, as well as the types of food, their ingested quantities and/or the customized other items of their preference, thus working on the principle of food autonomy [70]. The “10 Steps to Healthy Eating” are key points summarized in the last chapter of the “Dietary Guidelines for the Brazilian Population”, consisting of practical, easy, and economically accessible strategies to be applied in the participants’ day-to-day life [25].

#### 3.2.4. Follow-Up

Finally, in the follow-up period (3 months after the completion of the 12th month of data collection), the participants did not receive any nutritional aid. In the 15th month, the questionnaires from previous meetings were reapplied, along with the collection of the food diaries, aiming to detect the stability of behavior and post-intervention feeding.

#### 3.2.5. Anthropometric Evaluation

Weight (in kilograms/kg) and height (in centimeters/cm) were measured by the Digital Anthropometric Scale Welmy W200a^®^, (São Paulo, São Paulo/Brazil) with Estadiometer Welmy^®^, (São Paulo, São Paulo/Brazil) attached to the equipment. Prior to the consultation, patients were instructed to attend the meeting wearing light clothes and without having voluminous meals. For the measurements, the removal of shoes was requested, as well as the emptying of the urinary bladder. Sequentially, the individual was asked to step onto the scale and position themselves in opposition to the digital monitor, that is, with the spine flush with the rod of the stadiometer, in an orthostatic posture (looking at the horizon and expanded thoracic box). The data were collected in the morning, aiming to mitigate any type of water body oscillation recurring throughout the day [71]. The BMI calculation was expressed by the formula of weight (kg) divided by the square of height (m)^2^, with participants being considered to fall into the categories of underweight (<18.5 kg/m^2^); eutrophic (18.5–24.9 kg/m^2^); overweight (25.0–29.9 kg/m^2^); obesity I (30.0–34.9 kg/m^2^); obesity II (35.0–39.9 kg/m^2^); or obesity III (≥40 kg/m^2^). For patients > 60 years old, the following classification was adopted: underweight (≤22 kg/m^2^); eutrophic (>22.0 <27.0 kg/m^2^); and obesity (≥27.0 kg/m^2^) [4,6,23].

The waist and hip circumference were measured using an Anthropometric Metric Tape Sanny^®^ of 2.0 m (São Paulo, São Paulo/Brazil). The waist circumference (in centimeters/cm) was measured by passing the tape around the midpoint between the last rib and the hip bone. This point was previously traced with a specific pen for anthropometry [71]. Th desired waist circumference values are below 80 cm for women and 94 cm for men, indicating a “low cardiovascular risk”. Values from 80 to 87 cm for women and 94 to 101 cm for men indicate a “high cardiovascular risk”. Values above 88 cm for women and 102 for men indicate a “very high cardiovascular risk” [4,6,23].

The waist/hip ratio was also calculated by dividing the value of the waist circumference by the hip circumference (in centimeters/cm). The hip circumference was measured by passing the tape around the widest perimeter in the hip region and the midpoint of the buttock [72]. Desired values are <0.85 for women and <0.9 for men, indicative of a low cardiovascular risk. Values above the limit are classified as a high risk for cardiovascular diseases [4,6,23]. All the data mentioned in this session were evaluated every 3 months over the 12 months and subsequently in the follow-up (15th month).

#### 3.2.6. Evaluation of Cardiovascular Parameters

Blood pressure (BP) values were measured by the Omron Control Digital^®^ equipment (Rio de Janeiro, Rio de Janeiro/Brazil), after 5 to 10 min at rest, with 3 readings on the RUA (Right Upper Arm) and LUA (Left Upper Arm) with a 1 min interval between measurements. For patients with BP values > 160/90 mmHg, a rest of 5 min was performed before taking another measurement. For patients with BP values > 200/100 mmHg, for which the equipment was not able to measure the pressure, a conventional measurement method was used via a Sphygmomanometer Nylon Premium Black^®^ (São Paulo, São Paulo/Brazil) and Black Double Stethoscope Solidor CX^®^ (São Paulo, São Paulo/Brazil). Individuals with SBP ≥ 140 mmHg and DBP ≥ 90 mmHg [3] were considered to have hypertension.

When office measurements were used, the diagnosis of Systemic Arterial Hypertension (SAH) was always validated by repeated measurements, under ideal conditions, on two or more medical visits at intervals of days or weeks; or more assertively, also taking measurements outside the office. A cuff of an appropriate size to the patient’s arm was used, about 2 to 3 cm above the antecubital fossa, centering the rubber bag over the brachial artery. The width of the rubber bag corresponded to 40% of the arm circumference and its length, involving at least 80% [72]. All the mentioned parameters were collected during the nutritional evaluation using the specific protocols for this [64,65]. The data on blood pressure and heart rate were evaluated every 3 months over the 12 months and subsequently in the follow-up (15th month).

#### 3.2.7. Evaluation of Laboratory Parameters

The analysis and collection of blood were carried out at the Clinical Analysis Laboratory of the Base Hospital-FUNFARME in São José do Rio Preto, São Paulo/Brazil. The collection was carried out by a trained Nursing Technician using a traditional venous puncture. The patients were fasting for a maximum of 12 h. The HbA1c and glucose were analyzed by Siemens^®^ equipment (Berlin/Germany).

For the diagnosis of diabetes, two dosages of fasting blood glucose ≥ 126 mg/dL after a minimum fast of 8 h or glycated hemoglobin ≥ 6.5% [2,3,23] were considered. The total cholesterol (TC), triglycerides (TGs), and HDL cholesterol were calculated by Roche cobas c501 equipment (Basel/Switzerland). The LDL cholesterol was estimated using the Friedewald Equation ([LDL] = (TC − HDL) − (TG/5). When the values of the triglycerides were above 400 mg/d, the LDL cholesterol was calculated by Roche cobas c501^®^ equipment (Basel/Switzerland) with Sekisu^®^ reagents (Beijing, China) [2,3,23]. Adequate levels of LDL cholesterol in healthy people should be below 130 mg/dL of blood and for people who present some risk condition, the levels should not exceed 70 mg/dL. The adequate levels of HDL cholesterol should be higher than 40 mg/dL of blood. The values of total cholesterol considered to be normal should be below 190 mg/dL, slightly high when they are between 200 and 239 mg/dL, and high when they are above 240 mg/dL. As for the values of the triglycerides, values below 130 mg/dL are desired [2,3,23].

All data were collected during the nutritional evaluations using the clinical evaluation protocol [64,65]. The laboratory data were evaluated every 3 months over the 12 months and subsequently in the follow-up (15th month).

#### 3.2.8. Evaluation of Medications

All medication data were collected during outpatient nutritional consultations for the intervention group and the control group, using the clinical evaluation protocol throughout the intervention from the 1st to the 12th month [64,65].

#### 3.2.9. Evaluation of Physical Exercises

The presence or absence of physical exercises was evaluated through conventional qualitative measurement/the survey of the presence or absence of active or sedentary individuals. Patients with levels equal to or greater than 5 trainings sessions per week, with a total duration of 250 min/week, were classified as “active (>5 sessions)/moderate (=5 sessions)”. Patients with levels equal to 3 trainings sessions per week, with a total duration of 150 min/week, were classified as “light”. Individuals below this recommendation (<3 sessions or zero) were considered to be “sedentary” [73]. All data were collected during the nutritional evaluation using the anthropometric and clinical evaluation protocols [64,65]. The exercise data were evaluated every 3 months over the 12 months. Note: Physical exercise is characterized as any activity performed under planning, in a determined period of time, with the purpose of improving health status and quality of life [73].

#### 3.2.10. COVID-19 Pandemic

COVID-19 infection was evaluated throughout the intervention from 1st to 12th months, using the metric “yes” or “no”. Individuals who reported “yes” were infected with COVID-19 at least once during the study and individuals who reported “no” were not infected with COVID-19 during the study. All data were collected during the nutritional evaluation using the specific protocols for this [64].

#### 3.2.11. Assessment of Eating Window and Sleep

This study also evaluated the habit of skipping breakfast, sleep duration, and nocturnal awakenings, as well as their possible influences on quantitative variables. However, due to the extensive nature of this study, these secondary results will be presented in another future scientific publication.

#### 3.2.12. Ethical Aspects

This research project was in accordance with the principles of the Declaration of Helsinki and Helsinki and approved by the Institutional Ethics Committee of the State Faculty of Medicine in São José do Rio Preto (FAMERP), São José do Rio Preto—São Paulo, Brazil—Human Research Ethics Committee, with its first approval on 18 July 2020 (CAAE: 33554520.0.0000.5415). It was also registered in Clinical Trials NCT06235762. All participants agreed to participate freely and voluntarily, signing the Informed Consent Term. Confidentiality and anonymity in relation to the content were guaranteed in order to preserve the identity of the interviewees. Written informed consent was also obtained from patients to publish this paper.

#### 3.2.13. Construction, Research Management, and Databases—REDCAP FAMERP/FUNFARME

The study data were collected, managed, and stored through the electronic data capture tools REDCap 14.0.9 hosted on REDCap—FUNFARME/FAMERP, the State Medical School of São José do Rio Preto—São José do Rio Preto, São Paulo, Brazil (https://redcap.hospitaldebase.com.br:44312, accessed from 16 August 2020 to 16 January 2024) [74,75]. The data presented in this study are available on request from the corresponding author. The data are not publicly available due to privacy.

#### 3.2.14. Statistical Analysis

Regarding the variables “sex”, “socioeconomic situation”, “race”, and “COVID-19 infection”, the absolute numbers of study participants per category were converted into a percentage relative to the total number of individuals who participated in the study and presented in descriptive tables and graphs, except for the variable of race, which was represented only in a table.

The other variables were subjected to the Shapiro–Wilk test for a normality check. The variables considered to have a normal distribution were “age”, “LDL cholesterol”, “waist circumference”, and “systolic blood pressure”. The other variables, “basal metabolic rate (BMR)”, “total energy expenditure (TEE)”, “total energy value (TEV)”, “fasting blood glucose”, “glycated hemoglobin”, “total cholesterol”, “HDL cholesterol”, “serum triglycerides”, “weight”, “BMI”, “waist-hip ratio”, “diastolic blood pressure”, and “heart rate”, showed non-normal distributions.

The variable “age”, obtained at the first consultation of the study, was compared between the control and intervention groups using an unpaired *t*-test. The variables “basal metabolic rate (BMR)”, “total energy expenditure (TEE)”, and “total energy value (TEV)”, also acquired at the first consultation of the study, were compared between the control and intervention groups using a Mann–Whitney test.

The other Gaussian variables, collected throughout the entire study, were compared between the groups for each time, and also within each group between the different times, using a two-way analysis of variance (ANOVA), repeated measures for intragroup (of treatment and time factors), followed by the Šídák multiple comparisons post hoc test. Meanwhile, the non-Gaussian variables, also acquired throughout the entire study, were compared between the groups for each time using the Kruskal–Wallis test followed by the Dunn’s multiple comparisons post hoc test, and within each group across different times using the Friedman test followed by the Dunn´s multiple comparisons post hoc test.

Data with normal distributions were presented as mean ± standard deviation (SD) and data with non-normal distributions as median ± interquartile ranges (IQR). The data referring to the aforementioned analyses were presented in descriptive tables and graphs. For all cases, α < 0.05 and *p* < 0.05 were adopted. The statistical analyses were performed using GraphPad Prism 9.0 ^®^ software [76].

## 4. Results

### 4.1. Quantitative Results

#### 4.1.1. Sociodemographic Data and Another Baseline Information

**Figure 2:**
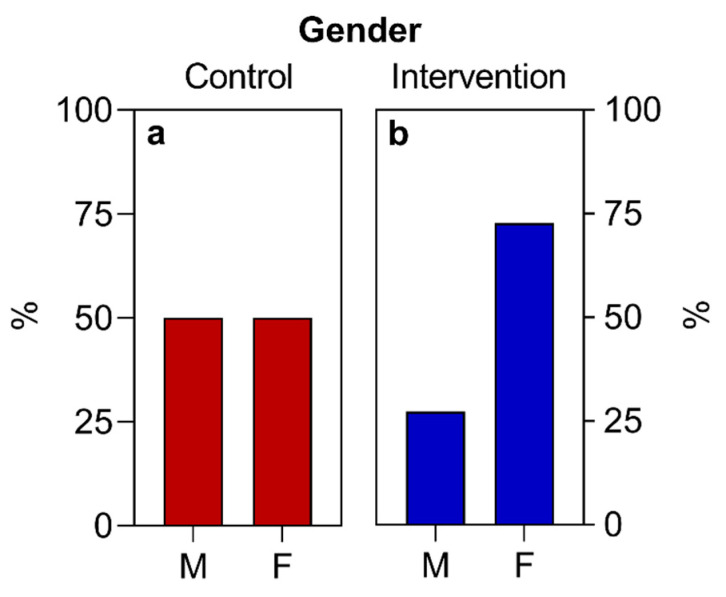
Gender of study participants. Description of the percentage of participants by gender in the control (**a**) and intervention (**b**) groups. M: male; F: female.

**Figure 3:**
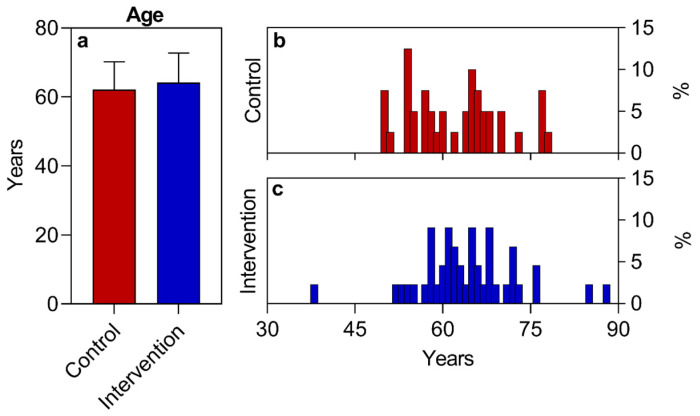
Age of study participants. Summarization of age by study group (control or intervention) (**a**) and descriptive histogram of the percentage of participants by age in the control (**b**) and intervention (**c**) groups. Data are presented as mean ± standard deviation. There is no significant difference between the age values presented by the control and intervention groups (unpaired *t*-test; *p* > 0.05).

**Figure 4:**
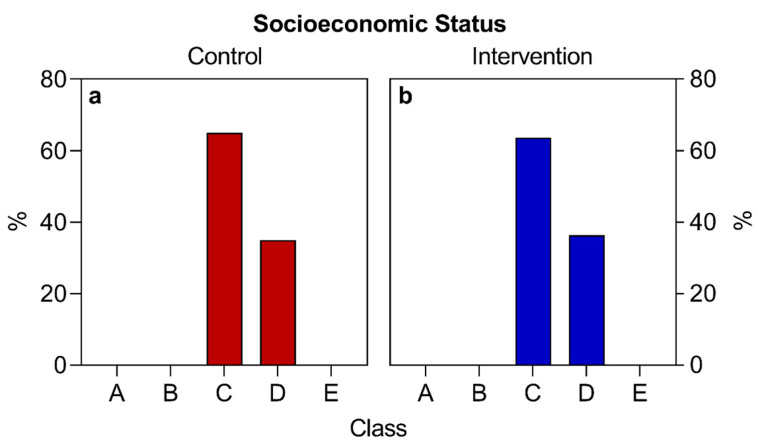
Socioeconomic status of study participants. Descriptive histogram of the percentage of participants by socioeconomic class in the control (**a**) and intervention (**b**) groups.

**Figure 5:**
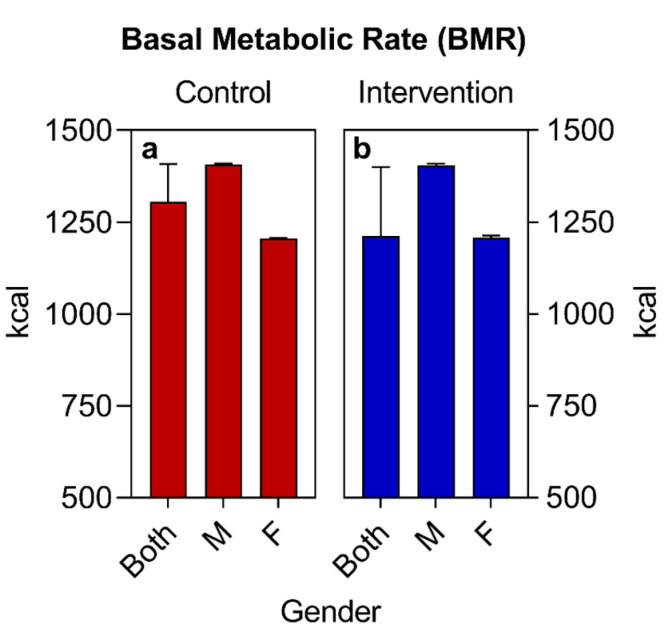
Basal metabolic rates (BMRs) of study participants. Summarization of BMRs for participants in the control (**a**) and intervention (**b**) groups. Data are presented as median ± interquartile range. There is no significant difference between the BMR values for both sexes presented by the control and intervention groups (Mann–Whitney test; *p* > 0.05).

**Figure 6:**
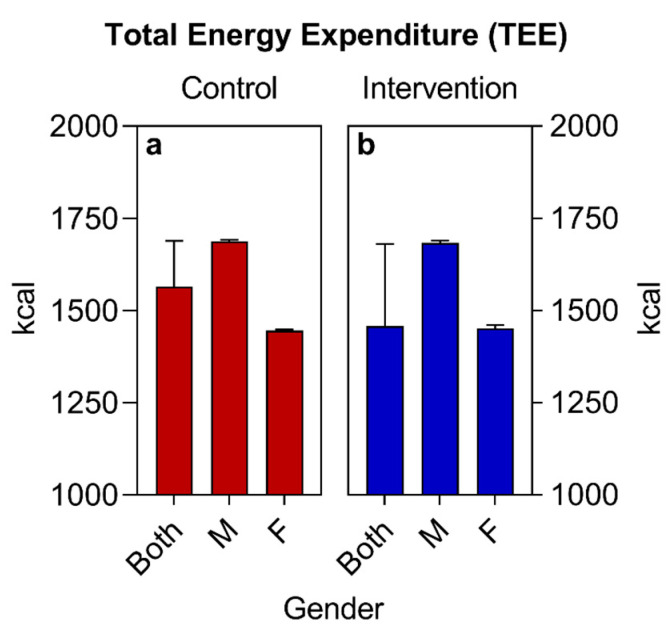
Total energy expenditures (TEEs) of study participants. Summarization of the TEEs for participants in the control (**a**) and intervention (**b**) groups. Data are presented as median ± interquartile range. There is no significant difference between the TEE values for both sexes presented by the control and intervention groups (Mann–Whitney test; *p* > 0.05).

**Figure 7:**
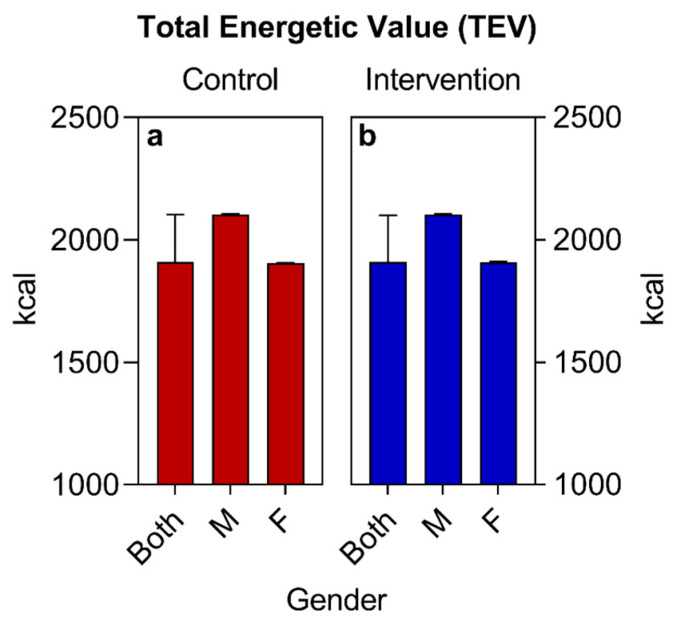
Total energetic values (TEVs) of study participants. Summarization of the TEVs for participants in the control (**a**) and intervention (**b**) groups. Data are presented as median ± interquartile range. There is no significant difference between the TEV values for both sexes presented by the control and intervention groups (Mann–Whitney test; *p* > 0.05).

Regarding the sociodemographic data and other information analyzed, similar values were observed for both groups at the beginning of the study (Table 2, Figure 2, Figure 3 and Figure 4), with the exception of the sex variable, in which a higher predominance of women (72.7%) was found in the intervention group compared to the control group (50%) (Figure 2). Regarding the other parameters, there were no significant statistical differences. The two groups had a predominance of participants with economic class C and D (Figure 4), also showing similarities in terms of average age (Figure 3), race, COVID-19 infection, BMR, TEE, and TEV (Table 2, Figure 5, Figure 6 and Figure 7). The data related to sex, age, race, and socioeconomic status were self-declared by the patients. Below is Table 2, with the comparative information of the samples at the baseline point and sequentially. Figure 2, Figure 3 and Figure 4 relate the sociodemographic data (gender, age, and socioeconomic status). Figure 5, Figure 6 and Figure 7 relate the BMR, TEE, and TEV data, respectively.

#### 4.1.2. General Lifestyle Data, Anthropometric, Biochemical, and Cardiovascular Parameters at Baseline

In the analysis of the anthropometric and biochemical data, similar values were observed in both groups at the beginning of the study, with no significant statistical differences detected, except for the value of diastolic blood pressure, which was slightly higher in the intervention group (*p* < 0.0464). Below is Table 3, illustrating the comparison between the samples in the baseline period.

#### 4.1.3. Comparison of Groups (Control and Intervention) with Their Own Metrics between Baseline and the 12th Month for Anthropometric, Biochemical, Cardiovascular Parameters, and General Lifestyle Data

Significant differences were observed in the anthropometric, biochemical, and cardiovascular data between the baseline period and 12 months in the intervention group, with a reduction in weight (*p* < 0.0001), BMI (*p* < 0.0001), WC (*p* < 0.0001), the WHR (*p* < 0.0001), FBG (*p* < 0.0001), HbA1c (*p* < 0.0001), LDL-C (*p* < 0.0001), TGs (*p* < 0.0001), SBP (*p* < 0.0001), DBP (*p* < 0.0001), HR (*p* = 0.0129), and an increase in HDL-C (*p* = 0.0105), except for TC (*p* > 0.9999). On the other hand, in the control group, there was a significant increase in FBG (*p* = 0.0028), HbA1c (*p* < 0.0001), weight (*p* < 0.0001), BMI (*p* < 0.0001), and the WHR (*p* = 0.0003). Regarding the other parameters, there was no significant difference for TGs (*p* > 0.9999), TC (*p* = 0.2326), LDL-C (*p* > 0.9999), HDL-C (*p* = 0.0818), WC (*p* = 0.0987), HR (*p* > 0.9999), SBP values (*p* = 0.9998), and DBP (*p* > 0.9999) in the baseline periods and after 12 months of observation. Below is Table 4, regarding the comparison of groups (control and intervention) with their own metrics between the baseline and the 12th month for the anthropometric, biochemical, and cardiovascular parameters.

#### 4.1.4. Longitudinal Comparison of the Intervention Group with the Control Group for Anthropometric, Biochemical, and Cardiovascular Parameters over 12 Months, Followed by 3 Months of Follow-Up (15th Month)

The comparison of the control group versus the intervention group showed a significant difference for all analyzed parameters (*p* < 0.05) in the period from first to the twelfth month. In the follow-up period (the twelfth to the fifteenth month), there was also a difference (*p* < 0.05), except for in BMI. The comparisons of the groups with their own times and between the groups from the first to the fifteenth month are illustrated in the following Figures, along with the description of each variable, as well as the contemplated results.

**Figure 8:**
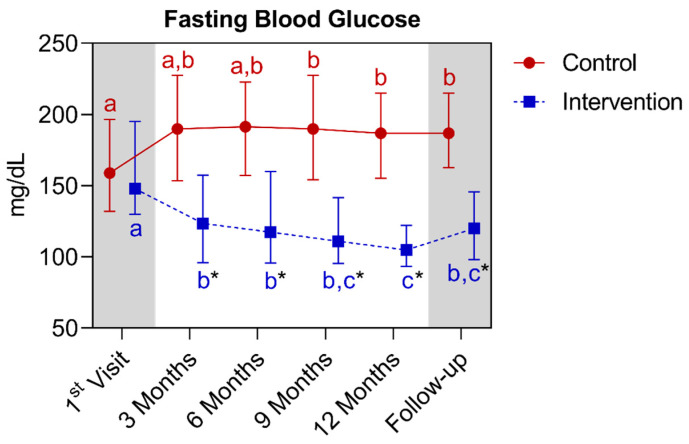
The fasting blood glucose of participants in the control and intervention groups over the study period. Data are presented as median ± interquartile range. Within each group (same data line), values that do not share at least one superscript letter have a significant difference between them (Friedman test; Dunn’s post hoc test; *p* < 0.05). Within the same period, an asterisk indicates a significant difference between the values of each group (Kruskal–Wallis test; Dunn’s post hoc test; *p* < 0.05).

Regarding the fasting blood glucose, significant differences were detected between the groups as early as the third month of intervention, extending until the follow-up period. As for the comparison between the control group with its own metrics over time, a significant increase was observed between the first and the ninth month, with continuity until the follow-up period. As for the intervention group, significant reductions were observed between the baseline and the third month, extending until the follow-up period. There was also a difference between the twelfth month in relation to first, third, and sixth month. Below is Figure 8, regarding the comparison of the fasting blood glucose values.

**Figure 9:**
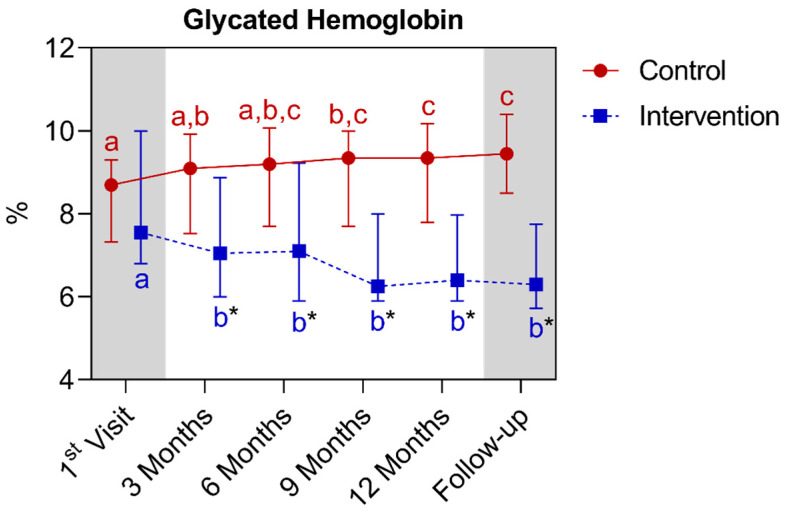
The percentage of glycated hemoglobin of participants in the control and intervention groups over the study period. Data are presented as median ± interquartile range. Within each group (same data line), values that do not share at least one superscript letter have a significant difference between them (Friedman test; Dunn’s post hoc test; *p* < 0.05). Within the same period, an asterisk indicates a significant difference between the values of each group (Kruskal–Wallis test; Dunn’s post hoc test; *p* < 0.05).

For the HbA1c analysis, statistically significant differences were also observed between the groups as early as the third month, continuing until the follow-up period. For the control group, when compared with their own metrics over time, a significant increase was observed between the baseline and the ninth month, extending until the follow-up period. A difference was noted between the twelfth and fifteenth month with the first and third months’ points. In the intervention group, a significant reduction was observed between the first and the third month, extending until the fifteenth month. Below is Figure 9, referring to the comparison of glycated hemoglobin values.

**Figure 10:**
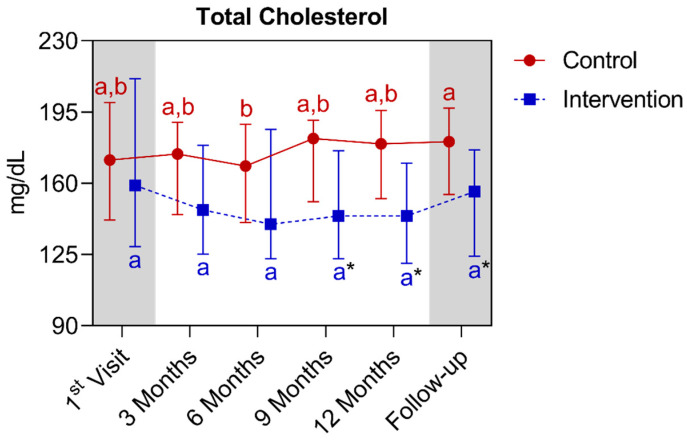
Total cholesterol of participants in the control and intervention groups over the study period. Data are presented as median ± interquartile range. Within each group (same data line), values that do not share at least one superscript letter have a significant difference between them (Friedman test; Dunn’s post hoc test; *p* < 0.05). Within the same period, an asterisk indicates a significant difference between the values of each group (Kruskal–Wallis test; Dunn’s post hoc test; *p* < 0.05).

Regarding the total cholesterol analysis, significant differences were observed between the groups from the ninth to the fifteenth month. In the comparison of the control group with its own metrics, a significant increase was only observed between the sixth and fifteenth months’ points. For the intervention group, there was no statistical difference among its own metrics over time. Below is Figure 10, which refers to the comparison of total cholesterol values.

**Figure 11:**
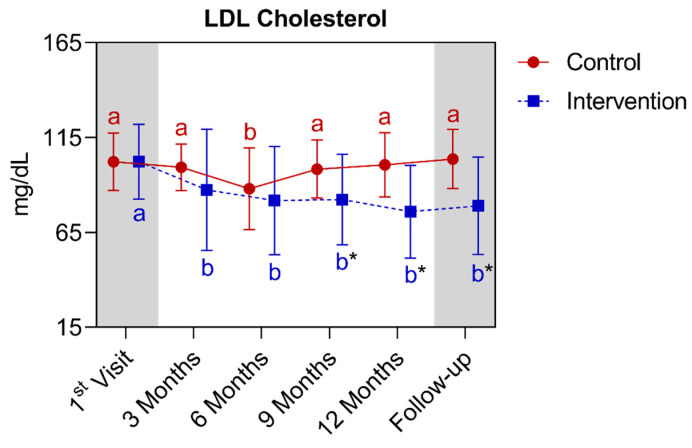
The LDL cholesterol values of participants in the control and intervention groups over the study period. Data are presented as mean ± standard deviation. Within each group (same data line), values that do not share at least one superscript letter have a significant difference between them. Within the same period, an asterisk indicates a significant difference between the values of each group [two-way ANOVA (repeated measures for intragroup); Šídák’s post hoc test; *p* < 0.05].

Regarding the LDL values, a statistically significant difference was observed between the groups starting from the ninth month, extending to the follow-up. In the comparison of the control group with its own metrics over time, an acute significant reduction was only observed in the sixth month compared to the other points analyzed. For the analysis of the intervention group, a significant reduction was detected between the time of the first and the third month, which extended until the follow-up. Below is Figure 11, referring to the comparison of LDL cholesterol values.

**Figure 12:**
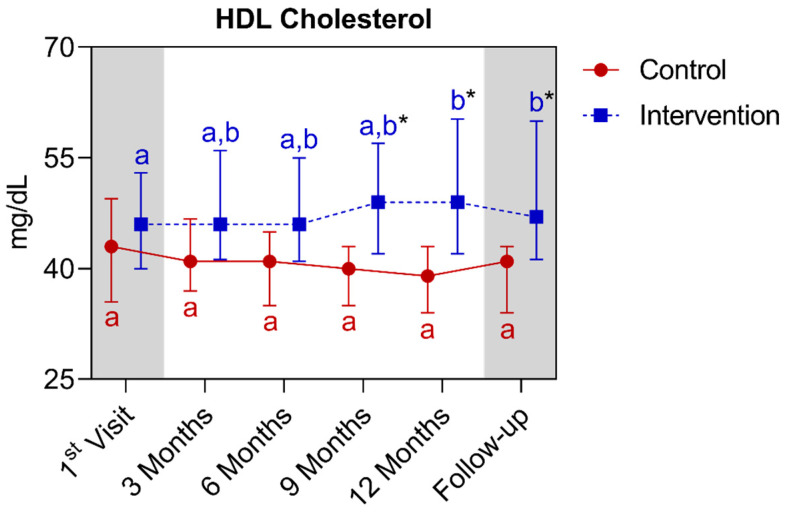
The HDL cholesterol values of participants in the control and intervention groups over the study period. Data are presented as median ± interquartile range. Within each group (same data line), values that do not share at least one superscript letter have a significant difference between them (Friedman test; Dunn’s post hoc test; *p* < 0.05). Within the same period, an asterisk indicates a significant difference between the values of each group (Kruskal–Wallis test; Dunn’s post hoc test; *p* < 0.05).

In the HDL-C analysis, a statistically significant difference was observed between the groups starting from the ninth month, extending until the fifteenth month. In the comparison of the control group with its own metrics over time, no significant differences were observed over time. Regarding the intervention group, a significant increase was observed between the baseline and the twelfth month, extending to the follow-up. Below is Figure 12, referring to the comparison of HDL cholesterol values.

**Figure 13:**
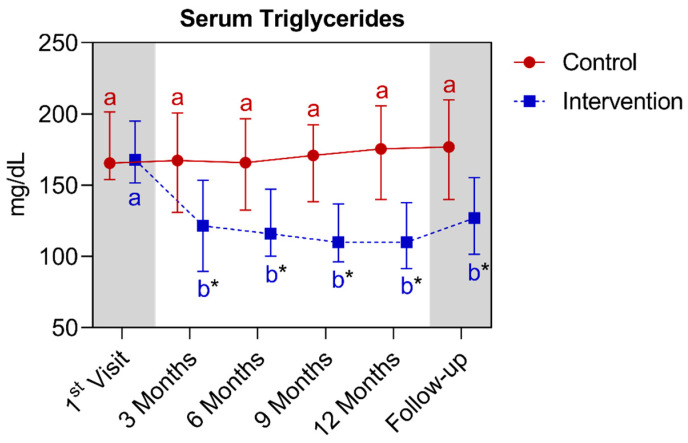
The serum triglycerides of participants in the control and intervention groups over the study period. Data are presented as median ± interquartile range. Within each group (same data line), values that do not share at least one superscript letter have a significant difference between them (Friedman test; Dunn’s post hoc test; *p* < 0.05). Within the same period, an asterisk indicates a significant difference between the values of each group (Kruskal–Wallis test; Dunn’s post hoc test; *p* < 0.05).

In the analysis of triglycerides, a statistically significant difference was observed between the groups starting from the third month, extending until the fifteenth month. In the comparison of the control group with its own metrics over time, no significant differences were observed over time. Regarding the intervention group, a significant reduction was observed as early as the third month, prolonging the results until the fifteenth month. Below is Figure 13, referring to the comparison of the triglyceride values.

**Figure 14:**
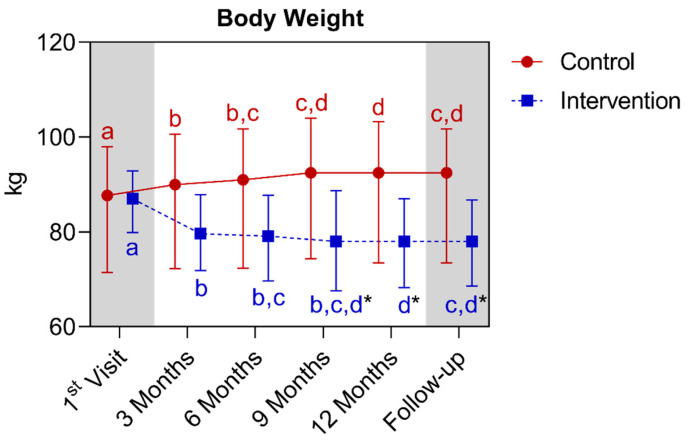
The body weight values of participants in the control and intervention groups over the study period. Data are presented as median ± interquartile range. Within each group (same data line), values that do not share at least one superscript letter have a significant difference between them (Friedman test; Dunn’s post hoc test; *p* < 0.05). Within the same period, an asterisk indicates a significant difference between the values of each group (Kruskal–Wallis test; Dunn’s post hoc test; *p* < 0.05).

In the weight analysis, a statistically significant difference was observed between the groups starting from the ninth month, extending until the fifteenth month. In the comparison of the control group with its own metrics over time, significant increases were observed between the baseline and the third month, extending until the follow-up period. It is noted that in the twelfth month there was a difference in relation to the baseline, third, and sixth month. Regarding the intervention group, a significant reduction was observed between the baseline and all other points analyzed. It is noted that the twelfth month differed from the first, third, and sixth month. Below is Figure 14, referring to the comparison of weight values.

**Figure 15:**
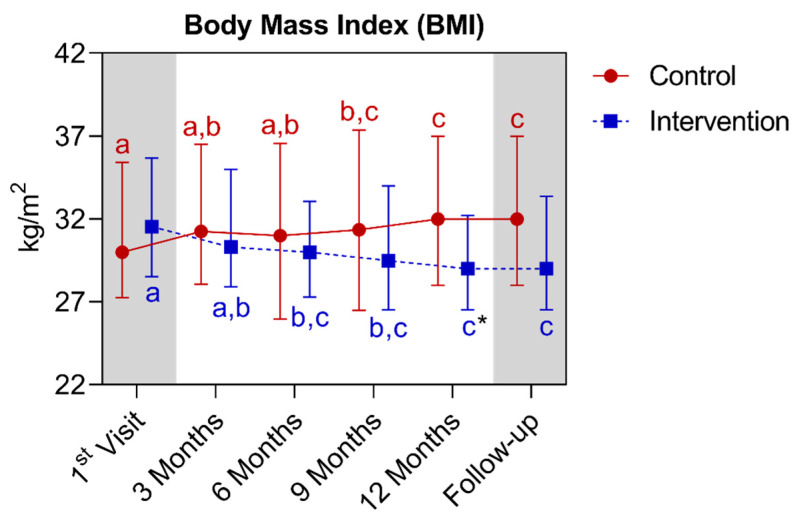
The BMIs of participants in the control and intervention groups over the study period. Data are presented as median ± interquartile range. Within each group (same data line), values that do not share at least one superscript letter have a significant difference between them (Friedman test; Dunn’s post hoc test; *p* < 0.05). Within the same period, an asterisk indicates a significant difference between the values of each group (Kruskal–Wallis test; Dunn’s post hoc test; *p* < 0.05).

In the BMI analysis, a statistically significant difference was observed between the groups only in the twelfth month. In the comparison of the control group with its own metrics over time, significant increases were observed between first and the ninth month, extending until the follow-up. A difference is noted between the twelfth and fifteenth month with the baseline and third, and sixth months’ points. Regarding the intervention group, a significant reduction was observed between first and the sixth month, extending until the fifteenth month. It is noted that in the twelfth and fifteenth month there was a difference between the first and the third month. Below is Figure 15, referring to the comparison of BMI values.

**Figure 16:**
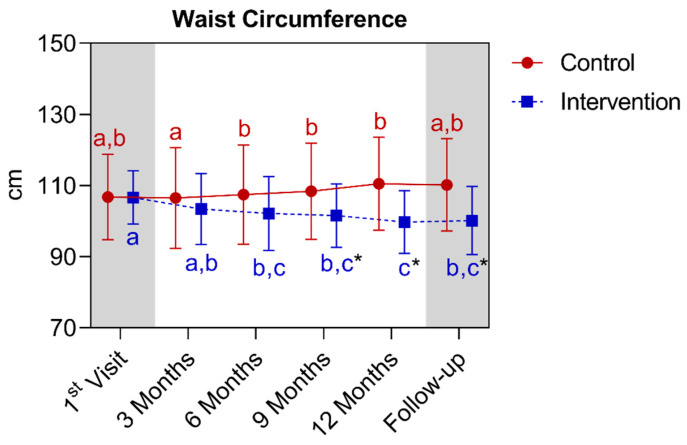
The waist circumferences of participants in the control and intervention groups over the study period. Data are presented as mean ± standard deviation. Within each group (same data line), values that do not share at least one superscript letter have a significant difference between them. Within the same period, an asterisk indicates a significant difference between the values of each group [two-way ANOVA (repeated measures for intragroup); Šídák’s post hoc test; *p* < 0.05].

In the analysis of waist circumferences, a statistically significant difference was observed between the groups starting from the ninth month, extending until the fifteenth month. In the comparison of the control group with its own metrics over time, significant increases were observed between the third and sixth month, extending until the twelfth month. Regarding the intervention group, a significant reduction was observed between the first and the sixth month, extending to the other analyzed moments and, subsequently, presenting a new reduction in the twelfth month. A difference is noted between the twelfth month and the baseline and the third month. Below is Figure 16, referring to the comparison of waist circumference values.

**Figure 17:**
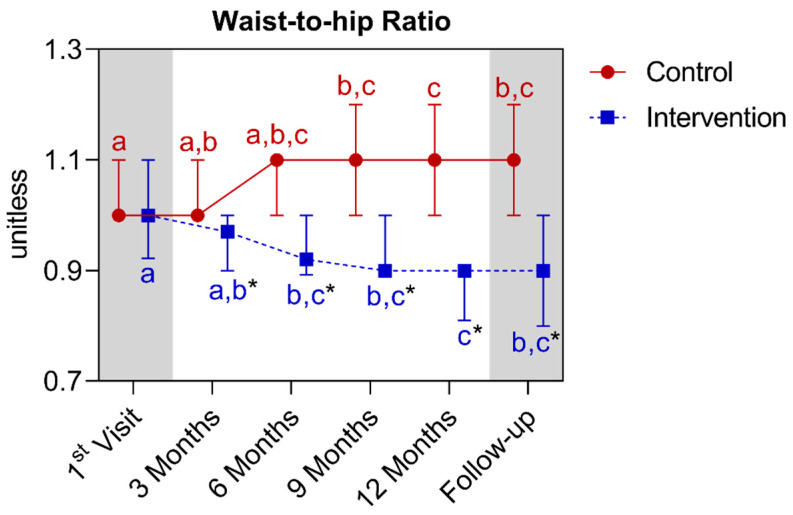
The waist-to-hip ratios of participants in the control and intervention groups over the study period. Data are presented as median ± interquartile range. Within each group (same data line), values that do not share at least one superscript letter have a significant difference between them (Friedman test; Dunn’s post hoc test; *p* < 0.05). Within the same period, an asterisk indicates a significant difference between the values of each group (Kruskal–Wallis test; Dunn’s post hoc test; *p* < 0.05).

In the waist-to-hip ratio analysis, a statistically significant difference was observed between the groups starting from the third month, extending until the fifteenth month. In the comparison of the control group with its own metrics over time, significant increases were observed between first and the ninth month, extending until the follow-up. It is noted that the twelfth month differed from the first and third month points. Regarding the intervention group, a significant reduction was observed between the first and the sixth month, extending until the follow-up. It is noted that the twelfth month differed from the first and third months’ points. Below is Figure 17, referring to the comparison of waist-to-hip ratio values.

**Figure 18:**
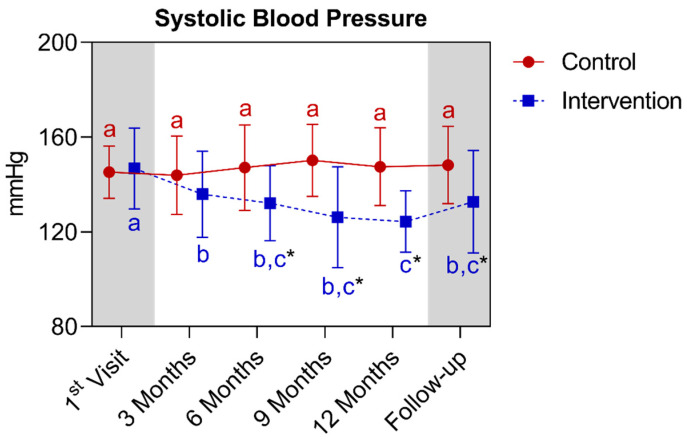
The systolic blood pressure values of the participants in the control and intervention groups over the study period. Data are presented as mean ± standard deviation. Within each group (same data line), values that do not share at least one superscript letter have a significant difference between them. Within the same period, an asterisk indicates a significant difference between the values of each group [two-way ANOVA (repeated measures for intragroup); Šídák’s post hoc test; *p* < 0.05].

In the analysis of systolic blood pressure, a statistically significant difference was observed between the groups starting from the sixth month, extending until the fifteenth month. When comparing the control group with its own metrics over time, no significant differences were detected. As for the intervention group, a significant reduction was observed between the baseline and the third month, which continued until the follow-up period. It is worth noting that the twelfth month differed from the baseline and the third month’s points. Below is Figure 18, which refers to the comparison of systolic blood pressure values.

**Figure 19:**
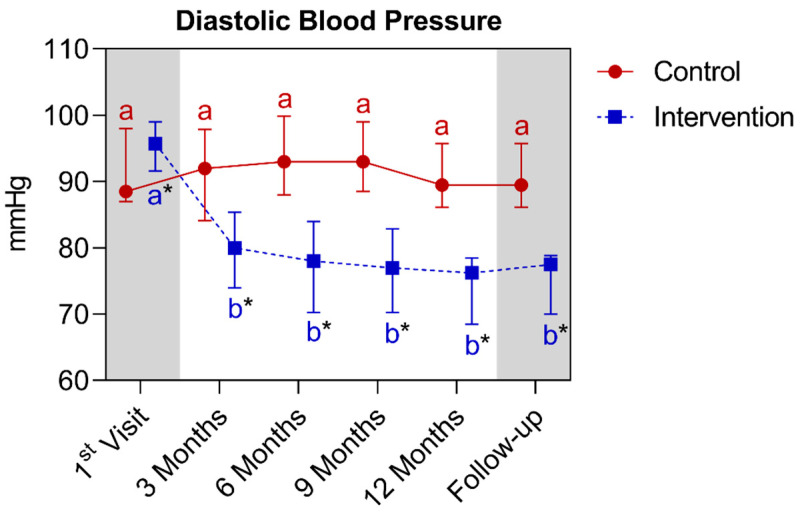
The diastolic blood pressure values of participants in the control and intervention groups over the study period. Data are presented as median ± interquartile range. Within each group (same data line), values that do not share at least one superscript letter have a significant difference between them (Friedman test; Dunn’s post hoc test; *p* < 0.05). Within the same period, an asterisk indicates a significant difference between the values of each group (Kruskal–Wallis test; Dunn’s post hoc test; *p* < 0.05).

In the analysis of diastolic blood pressure, a statistically significant difference was observed between the groups right from the baseline, given that the intervention group had slightly higher values than the control. It is worth noting that this difference was reversed as early as the third month, and this continued until the fifteenth month. When comparing the control group with its own metrics over time, no significant differences were observed. As for the intervention group, a significant reduction was observed between the baseline and the third month, which continued until the fifteenth month. Below is Figure 19, which refers to the comparison of diastolic blood pressure values.

**Figure 20:**
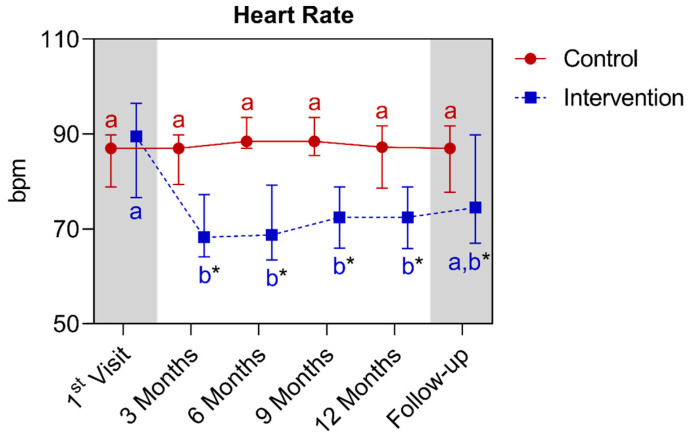
The heart rate values of participants in the control and intervention groups over the study period. Data are presented as median ± interquartile range. Within each group (same data line), values that do not share at least one superscript letter have a significant difference between them (Friedman test; Dunn’s post hoc test; *p* < 0.05). Within the same period, an asterisk indicates a significant difference between the values of each group (Kruskal–Wallis test; Dunn’s post hoc test; *p* < 0.05).

In the heart rate analysis, a statistically significant difference was observed between the groups starting from the third month, and this continued until the fifteenth month. When comparing the control group with its own metrics over time, no significant differences were observed. As for the intervention group, a significant reduction was observed between the baseline and the third month, which continued until the twelfth month. Below is Figure 20, which refers to the comparison of heart rate values.

### 4.2. Qualitative Results

#### 4.2.1. Diet and Eating Behavior

At the beginning of the intervention, the participants’ food diaries showed a high consumption of high-calorie foods such as sweets, cookies, biscuits, sodas, snacks (“coxinha”, “kibe”, breaded skewers, and pies), refined sugars, sodas, powdered drinks, nectar, bee honey, ready-made pizzas, frozen lasagnas, ice creams, processed meats (ham, mortadella, sausages, bacon, hot dogs, and frozen processed meat), instant noodles, and a high prevalence of delivery orders. In addition to their low purchasing power, patients reported a lack of interest in and management of financial resources. Many patients reported investing their resources in ultra-processed foods and eating their meals in restaurants (or ordering food from them), instead of spending on fresh or minimally processed products and preparing their own meals at home. Some common statements were recorded, such as “I spend on average BRL 50 to 100 reais on delivery every night”, “I’m not buying vegetables because the kg of tomato is BRL 8 reais”, “I don’t have time to cook so I buy ready-made food and/or go to restaurants”, and “when I’m very tired I don’t make lunch or dinner, I opt for a biscuit, snack or instant noodles so as not to be on an empty stomach”.

Various frequent “manifestations” of patients regarding the “high” prices of fruits and vegetables were reported, this being one of the main reasons reported for not consuming them frequently. Another frequently reported point was the “lack of time”, since many study participants have jobs and daily work taking up approximately 8 to 12 h, even though many of them reported being retired. A high prevalence of working women, providers and supporters of the family, was also verified in the study. More sensitive contexts, such as children and/or husbands involved with alcohol, the use of drugs and/or drug trafficking, theft, and prisoners, were also verified. Some patients did not have a family and/or relatives did not accompany them to the consultation, at some moments, the presence of neighbors, “godmothers/godfathers”, and sometimes the total absence of companions was observed. Abandonment was also observed at times, even in patients over 75 years old with frequent memory lapses and/or neurological comorbidities such as Alzheimer’s and Dementia, who would need a companion at the consultation.

On the other hand, throughout the nutritional intervention, an increase in the intake, offer, and exposure to fresh or minimally processed foods (vegetables, fruits, cereals, whole grains, tubers, legumes, meats, eggs, skimmed dairy, lean protein sources, aromatic herbs, spices, and others) and a reduction in the intake/purchase of ultra-processed foods in the intervention group patients was noticed. Another fact observed in this study was the increase in home-cooked meal preparation and consumption, as well as the greater consumption of “rice and beans” (a staple dish in Brazilian cuisine). However, this data was not verified in the control group.

#### 4.2.2. Regional Traditions, Culture, and Religion

In summary, most patients had traditional dietary characteristics of the Southeast region. It was also noticed that some patients had culinary habits from the Northeast culture (Bahia, Brazil), as they were born in the region and came to the state of São Paulo/Brazil in search of new opportunities. In these cases, the plan was properly adjusted to dietary preferences based on individuality (for example, the intake of couscous, “tapioca”, sweet potato, or cassava for breakfast). In addition, the habit of fasting during periods of religious celebration (Lent) and abstinence from certain foods (sweets, soda, and sugar) through “intentions, promises, and deliverances” was reported. Patients were properly advised about the dangers, contraindications, and worsening of glycemic control in relation to fasting, thus verifying a reduction of these over the course of the intervention. Occasionally, the following of fad diets and the use of other “healers” (lemon water, propolis, warm water with salt, vinegar, and others), aiming at glycemic control, was also verified. For this, patients were also properly advised about the absence of robust scientific evidence of such rituals/miraculous promises, especially involving T2D.

#### 4.2.3. Physical Activity

Regarding physical activities, participants revealed that they did not feel comfortable doing exercises. Some individuals pointed out that they did not like the environment of gyms or running tracks, as many looks were directed at their bodies. Others said they were unable, due to low mobility, joint pain, and a lack of “discipline, engagement, or motivation”, to exercise. Most participants were sedentary at the beginning of the study and remained so until the end of the intervention, both for the control group and the intervention group. Participants reported moving only with daily routine activities (cleaning, going to the supermarket, and walking the dog). During the pandemic, most patients remained in confinement and later returned to their daily routine activities and/or work.

#### 4.2.4. Medications

Regarding medications, all participants in both groups were using oral hypoglycemic agents such as metformin and/or glicazide. The use of other types of medications was also verified, such as antihypertensives (hydrochlorothiazide, furosemide, losartan, captopril, enalapril, atenolol, amlodipine, rampril, and others), statins (simvastatin, rosuvastatin, and atorvastatin), sodium levothyroxine, supplements (vitamin D, calcium, iron, and vitamin B12), and antidepressants (amitriptyline and sertraline). Most of the medications were prescribed in a medical consultation at the Hypertension or Endocrinology Outpatient Clinic, and were obtained at no cost at the popular SUS pharmacy.

Unfortunately, the level of education of the patients was low, making it difficult at times to understand their disease, the importance of the regular use of medications, basic concepts of nutrition, and others. Polypharmacy is another frequent issue in the routine of patients who often have lapses of memory or confusion with names, dosages, and medication times, also making adherence difficult, as well as the adequate control of blood glucose and blood pressure.

#### 4.2.5. Follow-Up

In the follow-up period (December 2022 to November 2023), it was noticed that some concepts passed at the beginning of the intervention were forgotten. A small relaxation of the intake of some ultra-processed foods with high caloric densities (cakes, sweets, biscuits, cookies, sodas, nectar, refreshments, instant noodles, processed meats, and sausages) and delivery orders were detected in the intervention group. It is noted that these points did not significantly alter the results, as the intake of these foods was not re-established daily.

## 5. Discussion

### 5.1. Sociodemographic and Qualitative Data

In terms of gender, the study predominantly featured women in the intervention group, unlike the control group, which had a more balanced male and female participation. The literature suggests that type 2 diabetes (T2D) can affect any individual, yet men are generally at a higher risk [1], particularly regarding insulin resistance and a predisposition to central adiposity due to androgenic factors [2]. The estimated prevalence of diabetes among women aged 20–79 is slightly lower than that of men (10.2% vs. 10.8%). As of 2021, there were 17.7 million more men with T2D than women [1]. Nonetheless, there has been a noticeable increase in cases among women in recent years, especially in those who are post-menopause, which is associated with a higher likelihood of central fat accumulation due to decreased estrogen levels, leading to increased insulin resistance [1,2].

Regarding socioeconomic aspects, there was a predominance of participants from socioeconomic classes C and D. This may be related to the fact that the interventions took place in the public service of the Diabetes and Hypertension Outpatient Clinic of the Unified Health System (SUS). Some evidence suggests that type 2 diabetes (T2D) can affect individuals across various economic classes [1], but recent epidemiological studies have shown an increasing predominance of T2D patients in lower socioeconomic classes, a trend also observed in individuals with obesity [4,5,6]. Research indicates that lower-income groups tend to make less informed choices regarding the nutritional quality of their food. Moreover, a lack of knowledge contributes to a loss of autonomy, of the ability to identify and understand the nutritional composition of food, its labeling, or even the best foods to manage their condition [77]. Other factors are related to geographic location; for instance, people with a lower socioeconomic status are often found in peripheral areas, hills, or regions far from commercial centers, thus hindering their access to markets, small producers, and other distribution centers for fresh and minimally processed foods [78]. More sensitive contexts with social and psychological vulnerabilities and long working hours are also more susceptible among the lower-income population, correlating with “compensation” or “emotional reward” as reasons for purchasing low-nutritional-quality foods [26,77,78].

It is noteworthy that in recent years, the “basic food basket” has undergone changes, with a reduction in minimally processed foods and an increase in ultra-processed foods [77,78]. Recent public policies are advocating for the reformulation of the basic basket, suggesting the inclusion of more fresh, minimally processed foods and culinary ingredients, while reducing the presence of ultra-processed foods [23,77]. This initiative is grounded in the Dietary Guidelines for the Brazilian Population, ensuring the right of every Brazilian to have access to nutritionally adequate foods [23,25,77]. Recent studies indicate that socioeconomic classes C and D spend the most on ultra-processed foods and delivery services, particularly after the pandemic [23,77,78]. Several reasons have been identified for these outcomes, such as their having limited access to knowledge and education, especially regarding their distorted perception of food “values,” and a lack of financial resource management [23,77]. It is observed that the little income earned is spent on foods of low nutritional quality, both through delivery services and wholesale purchases. In this context, it is incumbent upon society to develop strategies and public policies (campaigns, education, nutritional labeling, urban agriculture, community gardens, laws, RDCs, technology/app development, etc.) aiming to optimize access to nutritionally adequate food, information, and the “translation” of nutritional characteristics into playful label layouts, thereby facilitating the process of building food autonomy. Once informed and aware, individuals will have complete autonomy to make more informed choices to acquire foods of higher nutritional quality [23,25].

Within the age range of 38 to 88 years for the intervention group ($\overset{-}{X}$ ± SD = 64.2 ± 8.6) and 50 to 78 years for the control group ($\overset{-}{X}$ ± SD = 62.2 ± 8.0), a greater participation of adult individuals was noted in both groups. The literary findings generally show a higher incidence of type 2 diabetes (T2D) in the adult/elderly phase of life (40–80 years) [1], although this scenario is changing, with T2D also affecting younger individuals due to the parallel increase in other non-communicable chronic diseases (NCDs), particularly childhood and adolescent with obesity (<20 years) [2]. It is estimated that the prevalence of diabetes among those aged 75 to 79 was 24.0% in 2021, with a projected increase to 24.7% by 2045. The aging of the global population will lead to a growing proportion of overweight individuals with diabetes who are over the age of 60 [1].

Finally, regarding the variable of race, similar prevalences and proportions were observed in both groups for individuals who identified as white, brown, and black. The literature suggests that the prevalence of T2D is usually slightly higher in black populations [1]. This outcome may be hypothesized to relate to genetic factors, body structure, the distribution of adiposity, among other points, which largely do not establish mandatory causality, necessitating further studies on this topic [1,2]. Nonetheless, the findings of this research are significant, given the mixed-race nature of both sample groups, which is extremely relevant for representation in a multiethnic country like Brazil.

### 5.2. General Lifestyle Data, Anthropometric, Biochemical, and Cardiovascular Markers

The positive outcomes and successes of all variables obtained in the intervention group (compared to the control) may have been primarily due to the individualized nutritional management for each participant, aiming to care for metabolic, physiological, psychological, objective, and subjective aspects. References unanimously affirm the importance of personalizing the dietary plan, aiming for greater adherence to the plan and the long-term sustainability of results. The ideal diet is one adjusted to the peculiarities of each patient, respecting the principles of quantity, quality, harmony, and adequacy [10]. Furthermore, the prescribed dietary plans were based on the two nutritional strategies with the highest levels of evidence for treating type 2 diabetes (T2D), such as the Mediterranean diet and the DASH diet. Various epidemiological studies have reported the protective effect of these diets on metabolic disorders, chronic diseases, and mental health [2,10]. The main findings were related to the reduction of biochemical parameters (fasting glucose, HbA1c, LDL-C, and TC), of the incidence of T2D, cardiovascular risk, weight, and medication use [37,38,39,40,46].

In the current study, improvements in various markers were verified over 12 months and even in the follow-up, with the exception of the reduction of TC, where no significant difference was observed in the intervention group over the 12 months (when compared with itself). However, when comparing this variable between the groups (intervention vs. control), a significant difference was observed. The literature indicates that changes in TC are generally observed in the long term (>6 months), depending mainly on genetics and secondarily on lifestyle-related factors, such as smoking, alcohol, a sedentary lifestyle, and an unbalanced diet (foods with high caloric densities, that are rich in saturated fats, and low in fiber) [2,3,10].

Another interesting point observed in this study was the successful adaptation and reproduction of the Mediterranean and DASH dietary patterns to the Brazilian food culture. The key to such success would be the great food variability of these dietary patterns, without major severe restrictions. It is noted that in this study, foods with high nutritional qualities were prioritized, such as sources of carbohydrates (rice and beans as primary sources), mono- and polyunsaturated fats (canola, sunflower, and soy oils as primary sources), high fiber contents (seasonal fruits and vegetables), lean protein sources (various types of fish and skinless chicken), and low-fat dairy products (milk, yogurt, fresh “Minas” cheese, and ricotta) [57,58,59,60,61,62,63]. It is emphasized that at no point was there a need to overly restrict carbohydrates from the patients in this study, as has recently been spread by trendy diets such as the ketogenic, paleolithic, and intermittent fasting diets, all of which lack robust support (human clinical trials) to sustain the long-term management of type 2 diabetes (T2D).

A recent randomized clinical trial compared the ketogenic diet (~10–15% carbohydrates) vs. the Mediterranean diet (~35–40% carbohydrates) in patients with type 2 diabetes (T2D) over several months. Both diets included the incorporation of vegetables, the restriction of added sugars, and the limitation of refined grains. On the other hand, the consumption of legumes, fruits, and whole grains was encouraged only in the Mediterranean diet. The results showed a significant reduction in HbA1c for both diets; however, the ketogenic diet resulted in an increase in LDL-C, and a decrease in participants’ fiber intake, micronutrients, dehydration, worsened bowel function, and low long-term adherence [79]. These data are likely correlated with the severe carbohydrate restriction, lower fiber intake, and higher consumption of foods high in saturated fats, which is why the ketogenic diet is still not widely recommended by guidelines for the management of T2D [10].

Another study also indicated that the DASH diet could reduce levels of HbA1c, blood pressure, total cholesterol, and weight in patients with type 2 diabetes (T2D), but without significant differences in triglycerides [10]. Conversely, this study proved the diet to be potentially effective not only in improving triglycerides but also in various other markers that are not as well evidenced, such as HDL-C, the waist-to-hip ratio, and heart rate, especially in longitudinal follow-ups. It is known that an increase in HDL-C is related to a healthy lifestyle, but not necessarily to a lower cardiovascular risk. It was also noted that there was an improvement in LDL-C, a marker that indicates an increased risk of cardiovascular disease and the formation of atheroma plaques. This is very likely related to the high-fiber diet (fruits, vegetables, and legumes), the encouragement of mono/polyunsaturated fat intakes (canola, sunflower, and soy oils), and the discouragement of a saturated fat intake (coconut oil, pork lard, bacon, cracklings, among others). This finding is extremely relevant as it not only debunks the “miraculous” claims of rampant fad diets but also observed that other more economically accessible vegetable oils can also have positive effects on the lipid profile, similar to olive oil, which, unfortunately, is currently experiencing a gradual price increase in Brazil.

Dietary patterns that combine the DASH and Mediterranean diets, also known as the “MIND Diet,” have been associated with a lower risk of all-cause mortality in the general population due to the reduction of cardiovascular risk markers (TC, LDL-C, and TG) [80]. These dietary patterns include foods rich in mono- and polyunsaturated fatty acids, with the latter being mainly of the omega-3 type, showing robust evidence for the reduction of hypertriglyceridemia [81]. Some studies also indicate a positive effect of the MIND Diet on patients with neurodegenerative diseases, as omega-3 is related to the formation of neurotransmitters and the myelin sheaths of neurons [59]. In the current study, both groups included some patients with neurodegenerative conditions (Alzheimer’s and Parkinson’s). Unfortunately, there was no application of a specific instrument or test to assess whether there was indeed an improvement in cognitive processing or a delay in the progression of these types of diseases before and after the intervention.

### 5.3. Body Weight, Blood Glucose, and Blood Pressure

This research observed an average weight reduction of 11% over 12 months among participants in the intervention group, potentially one of the main factors contributing to the improvement of other anthropometric (BMI, waist circumference, and the waist-to-hip ratio), biochemical, and cardiovascular parameters. Initially, the diastolic blood pressure of the intervention group was slightly higher than that of the control group, a factor that did not affect the results, as during the study, this group experienced a significant progressive decrease from baseline to the 3rd month, extending up to the 15th month. These findings are interesting as they indeed illustrate the effectiveness of dietary interventions on weight loss and, consequently, in blood pressure management.

For patients with type 2 diabetes (T2D), a modest weight loss (5–7%) can improve blood sugar levels and reduce the need for oral hypoglycemic medications. A more substantial weight loss (>10%) is enough to lower their HbA1C, fasting blood sugar, the risk of mortality, and in some cases, lead to the remission of the disease. Unfortunately, maintaining these results over the long term continues to be a significant challenge. Medications (GLP-1 analogs and SGLT-2 inhibitors), bariatric surgery, mental health care, and physical exercise can complement dietary interventions, aiding in a 15% reduction in weight and sustaining long-term outcomes [2,3]. In this study, all potential factors that could indirectly affect body weight (medications and exercise) were carefully controlled, demonstrating that the nutritional intervention can also lead to significant long-term weight loss.

Longitudinal studies with durations of 12 months [82], 5 years [83], and 10 years [84] have been demonstrating that weight loss is relatively possible to sustain, provided there is continuous patient monitoring. It is noted that weight loss does not follow a straight line of constant reduction but rather resembles a “parabolic function,” with ups and downs, and a tendency to regain weight over time, yet still remaining slightly below the post-intervention baseline [2,3,17]. Various nutritional strategies have been studied over the years, and all of them have weight loss as a point of convergence. The importance of addressing excess weight is further reinforced by several studies showing that both obesity and type 2 diabetes (T2D) increase the risk of more severe infections from COVID-19 and other non-communicable diseases (NCDs) [2,3]. What will dictate the best type of diet will be the individuality of the patient, as well as their goals and preferences, thus increasing the possibilities, repertoires, prescriptions, and flexibility of the dietary plan [2,17]. In this sense, the measurement of height, weight, BMI, and waist circumference in annual or quarterly medical clinic visits should become mandatory, as these are simple, inexpensive, and potentially effective measures for weight control, thus enabling new considerations for treatment and the referral to a nutritionist [23].

Another factor that may contribute to the reduction of blood pressure, fluid retention, and consequently body weight is the excess intake of sodium. Dietary sodium recommendations are debated in the literature due to the individual variable response of blood pressure (BP) to sodium intake. Moreover, the effects of severe sodium restriction on BP in individuals who are already continuously using antihypertensive medications need to be better studied. A recent study observed that reducing sodium intake (<1500 mg) in the diet significantly lowered BP in most adults and those who are elderly, without resulting in excessive adverse events [62]. However, some guidelines suggest that its severe restriction can lead to hyponatremia and hydroelectrolytic disorders, especially in patients already using antihypertensives and diuretics [3,10,23]. Given the controversies over severe sodium restriction, its potential risks, and benefits, in this study, the daily intake recommendation was <2300 mg for all patients [10].

### 5.4. COVID-19 Pandemic

The COVID-19 lockdown clearly affected the lifestyle of the population and led to changes in their daily habits, with potential health consequences, especially for patients with T2D. A study published in 2020 showed an increase in the consumption of sugary foods, snacks, fast foods, food from delivery services, and a decrease in vegetable consumption during the COVID-19 pandemic. The data also showed a high percentage of physical inactivity before and after the lockdown [10,85,86,87]. These findings were also observed in this study, particularly regarding the sedentary behavior and the excessive consumption of ultra-processed foods by both groups at the baseline of the intervention (which took place right after the first lockdown). Access to fresh and minimally processed foods was reduced during the pandemic, as food transportation and accessibility were compromised. Concurrently, the delivery sector tripled its services and demands, thus favoring access to fast foods [10,87,88]. Later, a significant improvement in dietary habits was noticed for the intervention group, but they remained sedentary. For the control group, a worsening of dietary habits was observed throughout the project.

On the other hand, some studies have found opposing data, indicating that after the pandemic, some patients improved their awareness of their food intake and changed their lifestyle, especially after realizing that non-communicable diseases (NCDs) predispose them to COVID-19 infection, as well as contribute to the worsening and the sequelae of the disease [10,89]. Upon witnessing the deaths of at-risk groups and/or relatives, some patients changed their behavior and lifestyle to prevent infection [87,88,89,90]. New research indicates that in 2023, there was an increase in vegetable consumption by both adolescents and adults, although the consumption of ultra-processed foods and delivery services also increased (considering various social classes) [7].

### 5.5. Follow-Up

Despite a slight decline in the quality of the diets during this period, it was observed that there was no significant change in most of the positive results obtained in the intervention group (with the exception of BMI). This may be related to a good adherence to the dietary intervention and a “lifestyle turnaround” [2,17]. Even with a slight increase in the consumption of some low-nutritional-quality foods, fresh and minimally processed foods were still present in the patients’ daily lives, and the caloric deficit was maintained. According to the literature, various factors may be related to the instability of eating behavior in the post-intervention period. The first would be the natural human behavioral characteristic of returning to baseline habits, where weight regain and the worsening of markers can occur, especially due to increased appetite, the decreased palatability of food (getting tired of certain foods), changes in the microbiota, hormonal fluctuations, and genetic expressions during weight loss [91,92].

Another point to consider is that humans are complex, meaning they are constantly changing, and nothing is fixed. Clearly, the process of a lifestyle change involves ups and downs [93]. The consolidation of new habits depends on various criteria, such as time, action, consistency, motivation (internal and external), discipline, and pleasure. It is naturally acceptable not to follow a straight, progressive line of success in the execution of a lifestyle change [91,92,93]. Failures should be part of the process, especially to refine and enhance the learning of the patient and even that of the health professionals involved in the treatment of the patient with T2D. After all, diabetes and obesity are recurrent diseases, meaning they do not have a “cure,” and can only be controlled. This is simple but not very explored, as the body naturally makes physiological adaptations to enhance its evolutionary process of energy storage. In this sense, the evidence highlights the importance of flexibility in the dietary plan and avoiding excessive restrictions of carbohydrates, salt, and other items, as too much deprivation may not be so advantageous for glycemic control, palatability, and the adherence to the regimen [94,95].

Moreover, a good portion of the patients were over 60 years old, already experiencing memory lapses, reporting that they had “forgotten” or lost the prescribed dietary plan. According to the Guidelines, all interventions in geriatric patients require continuous surveillance, both by the patients and by health professionals, as there is a higher probability of them having low long-term dietary adherence, mediated by various problems, such as chewing, swallowing, low palatability, cognitive processing decline, memory lapses, reduced gait, and mobility [96,97].

### 5.6. Qualitative Aspects of Diet and Eating Behavior

Another fact observed in this study was the increase in the consumption of home-cooked meals. According to the Dietary Guidelines for the Brazilian Population, having meals at home enhances the patient’s relationship with food through the therapeutic act of cooking, promotes economic benefits, and optimizes the quality of the diet, as the patient knows exactly what they are consuming. An increase in the preparation and consumption of “rice and beans” was also observed in this study. “Rice with beans” is considered to be the Brazilian “base dish,” with an excellent nutritional composition, providing macro, micronutrients, fibers, and satiety throughout the day, especially for individuals living in food insecurity [25].

On the other hand, an increase in the consumption of ultra-processed foods was verified in the control group, that is, the consumption of hyper-palatable foods with high caloric densities, which have been pointed out to be one of the main factors for worsening the progressive weight gain of the population [88]. Patients also reported that they were practicing the mindful eating technique during meals, optimizing their awareness of their intake and food appreciation. Recent studies suggest that increased mindfulness and proper chewing during meals can contribute to greater satiety and can consequently impact the reduction of the total energy value ingested. Indirectly, such a technique can optimize weight loss, the BMI, and waist circumference, becoming a simple tool to be adopted in the context of public health [55]. However, there are still controversies about how much this could actually impact anthropometric parameters, and more studies are necessary [10].

### 5.7. Positive Points

- This study followed an interventionist approach and included a control group. The control group was composed of a sample that closely resembled the intervention group in most parameters during the baseline period.
- A follow-up was conducted, which is extremely relevant to assess the stability of eating behaviors post-intervention.
- There was a control of variables that could interfere with the results, such as medications and physical exercises.
- A completely individualized dietary plan, standardized to the Brazilian food culture (considering preferences and glycemic targets), was crafted and delivered at the time of the first consultation.
- A broad repertoire of food options and variability was provided, allowing for greater palatability and adherence, thus avoiding monotony, especially for patients with taste aversion and difficulty swallowing (>65 years).
- Participants’ nutritional management was adapted to their socioeconomic reality, enabling effective and accessible strategies. This aspect is interesting not only for the Brazilian public health system but also for other countries facing the dichotomy between rising obesity and diabetes rates, alongside high levels of food insecurity and malnutrition.
- There was constant contact with patients through phone and/or text messages or mobile video calls, maximizing the clarification of doubts.
- The data collection period was relatively long, that is, 3 years of follow-up, to fill out a 15-month spreadsheet, concurrent with the challenging presence of the global COVID-19 pandemic and lockdown.
- This work evaluated other qualitative aspects, which are not yet so elucidated in the literature, such as COVID-19 infection and its consequences for people with T2D.
- This study used a representative sample of a large part of the Brazilian population, both at the socioeconomic level (Classes C and D) and in racial aspects (white, brown, and black).
- This study provided a good literature review and update on the topic.

### 5.8. Limitations and Future Perspectives

- The COVID-19 pandemic reduced the flow of consultations during the second lockdown.
- The level of physical activity and patient engagement during the pandemic was also null.
- A shortage of fresh and minimally processed foods was observed in São José do Rio Preto city, SP, Brazil, especially in the first year of the pandemic (March 2020 to 2021).
- There was no random allocation or blinding of all those involved in the study (patients and researchers) to the distribution of groups.
- There was also little interaction with the patients in the control group, as they did not undergo nutritional assessment.
- The sample size was relatively small (*n* = 84). Unfortunately, it is very difficult to conduct longitudinal studies without reductions in sample size. This research, unfortunately, had some dropouts (patients stopped going to the clinic appointments) and deaths.
- The majority of participants in the intervention group were women, and the baseline DBP level was also slightly higher in this group compared to the control. However, it is noted that this difference was reversed by the 3rd month, extending until the 15th month. For future studies, greater attention is emphasized on obtaining groups of patients with proportional sex quantities and verifying the absence of differences between all parameters in the baseline period.
- Despite a large portion of the parameters showing similarity (with no statistical difference at the start of the study), the groups were not technically completely matched. Therefore, it cannot be stated that this is a clinical trial, but rather an experimental study. This is a common occurrence in experimental studies and does not necessarily invalidate the results, but it is an important factor to consider when interpreting them.
- The data analysis was not conducted by separating genders because the aim of this research was not to analyze the effect of the intervention on sex. Instead, it aimed to analyze the effect of the intervention on anthropometric, biochemical, and cardiovascular markers in individuals of both sexes and as many patients as possible. This was especially the case since a convenience sample was used, i.e., a sample of available patients interested in participating who were from the Hypertension and Endocrinology Outpatient Clinic. Separating the data analysis by gender would reduce the sample size to the point of altering the robustness of the results. In addition, the number of paired individuals was also similar (*n* = 40 control/*n* = 44 intervention). Dividing by gender would harm the analysis, decreasing the quality of the statistical power.
- There was a reversal of roles in seeking treatment, meaning the researcher approached the patients and invited them to participate in the study. This reversal of the search for treatment can hinder the patient’s interest and their appreciation of the intervention. In most successful clinical practice cases, the search is the opposite; the patient takes the initiative to seek out the professional and wants to change their lifestyle. The act of seeking help, the “action,” is the first step towards therapeutic optimization and is fundamental to establishing new eating habits.
- In future studies, it may be interesting to assess body masses (lean mass and body fat). Unfortunately, it was not possible to measure them, as the hypertension clinic did not have specific equipment for in-depth anthropometric assessment (a bioimpedance measure and an adipometer, for example). Moreover, most patients had abdominal obesity, which makes it difficult to measure skin folds. Another important point is that the participants were also using diuretics, which negatively interfere with hydration levels and, consequently, the accuracy of bioimpedance, for example.
- Additionally, it may be interesting to include the assessment of neck circumference and the waist (cm)/height (cm) ratio, as these are emerging measures [2] that have shown interesting results in patients with T2D and high cardiovascular risks [3], and being that these measures are simple and economically accessible for measurement at the ambulatory level of public health systems [23].
- Future studies may be able to increase the information derived from basic anthropometrics, with the use of allometric indices for waist circumference, A Body Shape Index (ABSI), and for hip circumference, the Hip Index (HI). For practical purposes, these indices are mutually independent of the BMI and can therefore be readily combined statistically [98,99].

## 6. Conclusions

Nutritional intervention was effective in improving the anthropometric, biochemical, and cardiovascular parameters in participants with type 2 diabetes (T2D) compared to those of the control group over a 12-month period and follow-up. The individualized dietary plan, drawing inspiration from the Mediterranean and DASH diets, demonstrated good adaptation to Brazilian food cultures and economic conditions. Importantly, the role of the nutritionist within an interdisciplinary team is crucial for maximizing nutritional recovery and addressing dietary inadequacies. Furthermore, economically accessible strategies, tailored to each patient’s individual needs, and continuous vigilance are essential for sustaining positive results in the long term. However, more clinical trials with appropriate randomization and group matching are necessary in order to optimize the level of evidence for longitudinal interventions.

## 7. Take-Home Message

- Personalized nutrition is a gold standard for the management of patients with type 2 diabetes mellitus (T2D). A single, universal strategy will never be the solution for the long-term control and prevention of this disease.
- It is possible to prescribe meal plans that combine taste and health, within contexts that involve socioeconomic vulnerabilities.
- The Mediterranean and DASH diets offer a wide food repertoire of foods and in recent years, they have been showing good results in adherence, in the longitudinal improvement of health parameters, and in their adaptation to other food cultures worldwide.
- Weight loss may not follow a straight, decreasing line, as it involves various complexities and challenges. Adjustments to the meal plan and constant monitoring are necessary to optimize long-term results.
- T2D is a complex disease, which affects different people, countries, and cultures. It does not have a specific color, address, or socioeconomic situation defined to it, that is, it can affect any individual, deserving constant attention from all public health systems.

## Figures and Tables

**Table 1:** Summary of the nutritional interventions.

Meetings	Objectives	Procedures
**1st meeting:** Initial Interview, evaluation, intervention, and data collection.	- Get to know and approach the participants. - Evaluate participants’ anthropometric, biochemical, cardiovascular, clinical, dietary, sociodemographic, and eating behavior parameters. - Analyze secondary eating behavior, sleep, and physical activity (collected only at the time of the 1st and 12th month). - Calculation, preparation, and delivery of the individualized food plan at the time of consultation. - Provide general complementary information to participants.	- Introductory informal chat. - Nutritional Anamnesis Protocol [64]. - Anthropometric, biochemical, and clinical evaluation protocol [65]. - Dietary evaluation protocol [64]. - Habitual reminder (food history) [64]. - Sociodemographic protocol [66]. - Provision: 1. Guidelines, information, and nutritional knowledge (APPENDIX 1 in Supplementary Materials); 2. Food plan (APPENDIX 2 in Supplementary Materials); 3. Food diary; 4. Delivery of the 10 steps for healthy eating (BRASIL, 2014) [25].
**Quarterly meetings:** Evaluation and data collection.	- Evaluate participants’ anthropometric, biochemical, cardiovascular, clinical, dietary, and sociodemographic parameters. - Analyze eating behavior, sleep, and physical activity (collected only at the time of the 1st and 12th month). - Provide general complementary information to participants.	- Nutritional Anamnesis protocol [64]. - Anthropometric, biochemical, and clinical evaluation protocol [65]. - Dietary evaluation protocol [64]. - Habitual reminder (food history) [64]. - Collection of the food diary and adjustment of the food plan when necessary.
**Follow-up**	- Analyze the stability of eating behavior and lifestyle changes post intervention. - Evaluate participants’ anthropometric, biochemical, cardiovascular, clinical, and dietary parameters.	- Feedback from participants about the continuity of interventions post-study. - Nutritional Anamnesis protocol [64]. - Anthropometric, biochemical, and clinical evaluation protocol [65]. - Dietary evaluation protocol [64]. - Habitual reminder (food history) [64]. - Collection of the food diary.

**Table 2:** Information about research participants from the control and intervention groups, obtained during the initial study visit.

	Control		Intervention		*p*
**Gender** **(number and percentage)**	**Male**	**Female**	**Male**	**Female**	n/a
	20 (50.0%)	20 (50.0%)	12 (27.3%)	32 (72.7%)	
**Age (years)** **(mean ± SD)**	62.2 ± 8.0		64.2 ± 8.6		0.2661
**Race**	** White Brown Black ** 10 (25%) 14 (35%) 16 (40%)		** White Brown Black ** 11 (25%) 15 (35%) 18(40%)		n/a
**Socioeconomic status (class) (number and percentage)**	**C**	**D**	**C**	**D**	n/a
	26 (65.0%)	14 (35.0%)	28 (63.6%)	16 (36.4%)	
**COVID-19 infection**	**No**	**Yes**	**No**	**Yes**	n/a
	12 (30.0%)	28 (70.0%)	12 (27.3%)	32 (72.7%)	
**BMR (kcal) (median ± IQR)**	1306 (1408–1204)		1212 (1400–1204)		0.4076
**TEE (kcal) (median ± IQR)**	1565 (1689–1446)		1458 (1680–1448)		0.5424
**TEV (kcal) (median ± IQR)**	1910 (2103–1904)		1911 (2100–1902)		0.2318

Non-Gaussian data expressed as median ± interquartile range (IQR); Gaussian data expressed as mean ± standard deviation (SD). BMR: basal metabolic rate; TEE: total energy expenditure; TEV: total energetic value; n/a: not applicable. There is no significant difference between the values of age, BMR, TEE, and TEV observed in the control and intervention groups (unpaired *t*-test for age; Mann–Whitney test for BMR, TEE, and TEV.; *p* > 0.05).

**Table 3:** The health indicator data of the research’s participants from the control and intervention groups, obtained during the initial study visit.

	Control			Intervention			*p*
	First Visit			First Visit			
**Exercise habits (number and percentage)**	**S** 40 (100%)	**L** 0 (0%)	**M/I** 0 (0%)	**S** 44 (100%)	**L** 0 (0%)	**M/I** 0 (0%)	n/a
**Fasting blood glucose (mg/dL) (median ± IQR)**	159.0 (196.5–132.0)			148.0 (195.3–130.0)			>0.9999
**Glycated hemoglobin (%) (median ± IQR)**	8.7 (9.3–7.3)			7.5 (10.0–6.8)			>0.9999
**Total cholesterol (mg/dL) (median ± IQR)**	171.5 (199.8–142.0)			159.0 (211.5–129.0)			>0.9999
**LDL cholesterol (mg/dL) (mean ± SD)**	102.4 ± 15.1			102.3 ± 19.7			>0.9999
**HDL cholesterol (mg/dL) (median ± IQR)**	43.0 (49.5–35.5)			46.0 (53.0–40.0)			>0.9999
**Serum triglycerides (mg/dL) (median ± IQR)**	165.5 (201.5–154.0)			168.0 (195.0–151.8)			>0.9999
**Body weight (kg) (median ± IQR)**	87.7 (98.0–71.5)			87.0 (92.9–79.9)			>0.9999
**BMI (kg/m^2^) (median ± IQR)**	30.0 (35.5–27.2)			31.5 (35.7–28.5)			>0.9999
**Waist circumference (cm) (mean ± SD)**	106.8 ± 12.0			106.7 ± 7.5			>0.9999
**Waist-to-hip ratio (unitless) (median ± IQR)**	1.0 (1.1–1.0)			1.0 (1.1–0.9)			>0.9999
**Systolic blood pressure (mmHg) (mean ± SD)**	145.3 ± 11.0			146.8 ± 17.1			0.9967
**Diastolic blood pressure (mmHg) (median ± IQR)**	88.5 (98.0–87.0)			95.7 (99.0–91.6) *			**0.0464**
**Heart rate (bpm) (median ± IQR)**	87.0 (89.9–78.9)			89.5 (96.5–76.6)			>0.9999

Non-parametric data expressed as median ± interquartile range (IQR); Parametric data expressed as mean ± standard deviation (SD). S: sedentary; L: light exercises; M/I: moderate or intense exercises; n/a: not applicable. An asterisk indicates a significant difference between values observed in the control group and the intervention group at the first visit (Kruskal–Wallis test followed by Dunn’s post hoc test for non-parametric data; two-way ANOVA followed by Šídák’s post hoc test for parametric data; *p* < 0.05).

**Table 4:** Health indicator data of research participants from the control and intervention groups, obtained at the initial visit and at the twelfth month.

	Control							Intervention						
	First Visit			Twelfth Month			*p*	First Visit			Twelfth Month			*p*
**Exercise habits (number and percentage)**	**S** 40 (100%)	**L** 0 (0%)	**M/I** 0 (0%)	**S** 40 (100%)	**L** 0 (0%)	**M/I** 0 (0%)	n/a	**S** 44 (100%)	**L** 0 (0%)	**M/I** 0 (0%)	**S** 44 (100%)	**L** 0 (0%)	**M/I** 0 (0%)	n/a
**Fasting blood glucose (mg/dL) (median ± IQR)**	159.0 (196.5–132.0)			187.0 (215.9–155.3) *			**0.0028**	148.0 (195.3–130.0)			105.0 (122.3–93.5) *			**<0.0001**
**Glycated hemoglobin (%) (median ± IQR)**	8.7 (9.3–7.3)			9.3 (10.2–7.8) *			**<0.0001**	7.5 (10.0–6.8)			6.4 (8.0–5.9) *			**<0.0001**
**Total cholesterol (mg/dL) (median ± IQR)**	171.5 (199.8–142.0)			179.5 (195.8–152.5)			0.2326	159.0 (211.5–129.0)			144.0 (170.0–120.8)			>0.9999
**LDL cholesterol (mg/dL) (mean ± SD)**	102.4 ± 15.1			100.7 ± 16.8			>0.9999	102.3 ± 19.7			76.0 ± 24.4 *			**<0.0001**
**HDL cholesterol (mg/dL) (median ± IQR)**	43.0 (49.5–35.5)			39.0 (43.0–34.0)			0.0818	46.0 (53.0–40.0)			49.0 (60.2–42.0) *			**0.0105**
**Serum triglycerides (mg/dL) (median ± IQR)**	165.5 (201.5–154.0)			175.5 (205.8–140.0)			>0.9999	168.0 (195.0–151.8)			110.0 (137.8–91.5) *			**<0.0001**
**Body weight (kg) (median ± IQR)**	87.7 (98.0–71.5)			92.5 (103.3–73.5) *			**<0.0001**	87.0 (92.9–79.9)			78.0 (87.0–68.2) *			**<0.0001**
**BMI (kg/m^2^) (median ± IQR)**	30.0 (35.5–27.2)			32.0 (37.0–28.0) *			**<0.0001**	31.5 (35.7–28.5)			29.0 (32.3–26.6) *			**<0.0001**
**Waist circumference (cm) (mean ± SD)**	106.8 ± 12.0			110.6 ± 13.1			0.0987	106.7 ± 7.5			99.7 ± 8.8 *			**<0.0001**
**Waist-to-hip ratio (unitless) (median ± IQR)**	1.0 (1.1–1.0)			1.0 (1.2–1.0) *			**0.0003**	1.0 (1.1–0.9)			0.9 (0.9–0.8) *			**<0.0001**
**Systolic blood pressure (mmHg) (mean ± SD)**	145.3 ± 11.0			147.6 ± 16.4			0.9998	146.8 ± 17.1			124.4 ± 13.0 *			**<0.0001**
**Diastolic blood pressure (mmHg) (median ± IQR)**	88.5 (98.0–87.0)			89.5 (95.7–86.1)			>0.9999	95.7 (99.0–91.6)			76.2 (78.5–68.5) *			**<0.0001**
**Heart rate (bpm) (median ± IQR)**	87.0 (89.9–78.9)			87.2 (91.7–78.6)			>0.9999	89.5 (96.5–76.6)			72.5 (78.9–65.9) *			**0.0129**

Non-normal data expressed as median ± interquartile range (IQR); Normal data expressed as mean ± standard deviation (SD). S: sedentary; L: light exercises; M/I: moderate or intense exercises; n/a: not applicable. Within each group (control or intervention), an asterisk indicates a significant difference between values observed at the first visit and the twelfth month (Friedman test followed by Dunn’s post hoc test for non-parametric data; two-way repeated measures ANOVA followed by Šídák’s post hoc test for parametric data; *p* < 0.05).

## Data Availability

Study data were collected and managed using REDCap 14.0.9 electronic data capture tools hosted at REDCap—FUNFARME/FAMERP (from the State Faculty of Medicine) [10,11]. The data presented in this study are available on request from the corresponding author. The data are not publicly available due to privacy.

## Supplementary Materials

The following supporting information can be downloaded at: https://www.mdpi.com/article/10.3390/nu16091378/s1, APPENDIX 1; APPENDIX 2.
