# Supplementary material for: The Impact of a Nutritional Intervention on Glycemic Control and Cardiovascular Risk Markers in Type 2 Diabetes
Source: Nutrients. 2024 May 1;16(9):1378. doi: 10.3390/nu16091378 (PMC11085322; doi:10.3390/nu16091378)
Supplement: Supplementary file 1 [file nutrients-16-01378-s001.zip › nutrients-2971681-supplementary.pdf]

SUPPLEMENTARY MATERIALS

1. APPENDIX 1

NUTRITIONAL AND GENERAL GUIDELINES FOR PARTICIPANTS WITH TYPE 2 DIABETES

***PRIORITIZE:**

-Begin meals with a salad or cooked vegetables [lettuce, tomato, cucumber, cabbage, chayote, zucchini, carrot, okra, eggplant, etc.]. Follow with a serving of lean protein [fish, skinless chicken, lean pork and beef cuts (eventuality), eggs, or textured soy protein (for vegetarians)]. Finally, consume your carbohydrate source [rice, beans, potatoes, sweet potatoes, cassava, pasta, corn, yams, etc.]. This sequence helps reduce post-meal blood sugar spikes.

-Incorporate fiber-rich foods into your daily routine (whole grains, legumes, vegetables, and fruits).

-Choose low-fat dairy products: milk, yogurt, curd, fresh "Minas" cheese, and ricotta. Note: Fresh Minas cheese is a traditional cheese produced in the region of Minas Gerais, SP, Brazil which was later expanded throughout the country and is now widely consumed by all. It resembles the taste of Italian low fat mozzarella cheese.

-Use canola, sunflower, or soy oil for cooking and seasoning.

-Include more fish in your meals, such as panga, hake, whiting, dogfish, sardines, tuna, tilapia, etc. (rich in omega-3).

-Choose lean meats: chicken breast, lean pork (pork loin and pork tenderloin), or lean beef (rump, round, muscle, tenderloin, and chuck center).

-When necessary, use artificial sweeteners in moderation instead of sugar (sucralose, stevia, acesulfame K, saccharin, and sodium cyclamate).

-Create your own herb salt for seasoning! Ingredients: one coffee cup of salt, oregano, rosemary, and basil. Blend everything and store in a sealed container.

-Aim for at least 4 meals a day.

-Gradually replace ultra-processed and packaged foods with fresh, whole foods.

-Practice Mindful Eating techniques: 1-Disconnect from your surroundings when seated at the table. 2-Avoid using your phone, TV, or radio during meals. 3-Savor the moment, noticing the aromas, temperature, and texture of the food. 4-Use your non-dominant hand to hold the fork (to prevent eating too quickly). 5-Chew your food thoroughly before swallowing, calmly and without haste. 6-Feel the texture, colors, and flavors of the food. 7-Control your breathing to reduce anxiety before eating. 8-Enjoy your food without judgment. 9-Develop the practice of being fully attentive to your food and bodily reactions during meals. 10-Rate your hunger and fullness before and after meals on a scale of 0 to 10.

-Engage in daily physical activity! Post-meal walks are also beneficial for reducing post-prandial blood sugar levels.

-Prefer daytime eating windows (7 am - 8 pm) in line with your biological circadian rhythm.

-Practice good sleep hygiene: maintain consistent sleep and wake times; avoid stimulant beverages (caffeine, chocolate, alcoholic drinks, black tea, green tea, and sodas) at least six hours before bedtime; don't eat heavy meals for dinner; exercise regularly, allowing at least four hours before bedtime; stay away from screens like phones, TVs, and tablets in bed at least an hour before sleeping. Aim for a total sleep duration of 8 hours per day.

***AVOID:**

-Saturated fats (poultry skin, fatty cuts of beef and pork, coconut oil, coconut milk, cream, butter, bacon, lard, whole milk, palm oil, MCT oil, etc.).

-Sugars (refined sugar, brown sugar, coconut sugar, honey, molasses, jaggery, ice cream, jellies, candied fruits, jams, etc.).

-Be cautious with excess salt (whether it's Himalayan, black, fleur de sel, light, etc.). Use only the necessary amount recommended by your nutritionist (5g of salt per day/ 5 salt sachets per day).

-Refined cereals (candies, sweets, cookies, cakes, pastries, pies, etc.).

10.2. APPENDIX 2

EXAMPLE OF A DIET PLAN PRESCRIBED TO PARTICIPANTS WITH TYPE 2 DIABETES

This is a model of the diet plan prescribed to participants, with suggestions for food repertoires, and schedules. It is important to remember that the diets were individualized and adjusted to the clinical case of each patient. The food repertoires included in this diet plan were inspired by the Mediterranean and DASH diets, and were duly adapted to the Brazilian dietary standard, regionalism, and the socioeconomic level of the SUS (Brazilian Public Health System) patients.

BREAKFAST – 7:00 AM:

Beverages:

- 1 medium cup (150ml) of coffee or tea without sugar (if necessary, sweeten with a few drops of sweetener)

Cereals (Carbohydrate Sources):

- 1 medium slice (25g) of whole wheat bread

  OR

- 1 small unit (25g) of French bread without the crumb

  OR

- 1 small unit (35g) of tapioca/couscous (2.5 level tablespoons)

  OR

- 2 units (20g) of whole grain or multigrain toast

  OR

- 4 units (20g) of whole grain rice cracker

Good Fat Source:

- 1 tablespoon (20g) of light ricotta cream (or light cream cheese or mashed avocado with salt and herbs)

Protein Source:

- 3 medium slices (60g) of light Minas cheese (or ricotta)

  OR

- 2 large units (120g) of eggs (boiled, poached, scrambled, omelet)

OR

- 3 tablespoons (50g) of shredded chicken with 1 tablespoon (10g) of light ricotta cream/light cream cheese/mashed avocado with salt and herbs.

Fruits (Source of Carbohydrates and Fibers):

- 1 medium slice (100g) of papaya, pineapple, melon, watermelon, or mango

OR

- 1 medium unit (100g) of tangerine or orange

OR

- 1 small unit (80g) of apple, pear, banana, or guava

OR

- 10 units (120g) of strawberry

OR

- 8 units (100g) of grape

OR

- 1 unit (100g) of any seasonal fruit of your choice (within appropriate portions)

Nuts and Seeds (Good Fat Source):

- 1 teaspoon (5g) of sunflower seeds, pumpkin seeds, sesame seeds, chia seeds, flax seeds, etc.

Spices and Aromatic Herbs:

- 1 coffee spoon (3g) of cinnamon to sprinkle on fruits.
- 1 coffee spoon (3g) of turmeric, black pepper, and aromatic herbs to season protein sources.

<u>MID-MORNING SNACK – 10:00 AM (CONSUME ONLY IF YOU ARE HUNGRY):</u>

Fruits (Source of Carbohydrates and Fibers):

- 1 medium slice (100g) of papaya, pineapple, melon, watermelon, or mango

OR

- 1 medium unit (100g) of tangerine or orange

OR

- 1 small unit (80g) of apple, pear, banana, or guava

  OR

- 10 units (120g) of strawberry

  OR

- 8 units (100g) of grape

  OR

- 1 unit (100g) of any seasonal fruit of your choice (within appropriate portions)

Nuts or Seeds (Good Fat Source):

- 2 tablespoons (20g) of a mix of nuts (example: peanuts, almonds, Brazil nuts, walnuts, etc.)

  OR

- 1 tablespoon (10g) of sunflower seeds, pumpkin seeds, sesame seeds, chia seeds, flax seeds, etc.

Spices:

- 1 coffee spoon (3g) of cinnamon to sprinkle on fruits

<u>LUNCH – 12:30 PM:</u>

Vegetables:

- 1 dessert plate full (200g) of raw and/or cooked vegetables (examples: lettuce, arugula, tomato, carrot, cucumber, chayote, broccoli, zucchini, eggplant, beetroot, okra, jiló, etc.)

Good Fat Source:

- 2 tablespoons (20g) of vinaigrette sauce (examples: tomato, onion, soy/canola/sunflower oil, lemon or vinegar, aromatic herbs, turmeric, pepper, and a pinch of salt)

  OR

- 1 tablespoon of canola/sunflower/soy oil + ½ lemon juice or vinegar, aromatic herbs, spices, and a pinch of salt

Protein Source:

- 1 medium fillet (120g) of cooked or grilled chicken breast (or thigh without skin and fat)

OR

- 1 medium fillet (120g) of cooked or baked fish (sardine, panga, hake, merluza, shark, tilapia, tuna, etc.)

  OR

- 1 medium fillet (110g) of lean beef cooked or grilled (top round, eye round, bottom round, shank, tenderloin, chuck center, and rump) - Option for twice a week

  OR

- 1 medium fillet (110g) of lean pork cooked or grilled (pork tenderloin and loin)

  OR

- 2 units (120g) of eggs (boiled, poached, scrambled, omelet) - If opting for eggs at breakfast, avoid repeating at lunch/dinner

  OR

- 4 medium slices (80g) of light Minas cheese or ricotta

  OR

- 4 tablespoons (100g) of sautéed textured soy protein (VEGAN OPTION)

  OR

- 4 tablespoons (100g) of cooked legumes (carioca beans, black-eyed peas, chickpeas, lentils, and peas) (VEGAN OPTION)

Cereals and Tubers (Carbohydrate Source):

- 3 level tablespoons (80g) of cooked rice (or cooked cassava or cooked parsley or cooked sweet potato or cooked corn or cooked pasta)

  OR

- 4 level tablespoons (100g) of cooked English potato

  OR

- 5 level tablespoons (120g) of cooked kabocha squash/pumpkin

Legumes (Source of Carbohydrates, Fibers, and Proteins):

- 1 medium ladle (80g) of cooked carioca beans (or black beans or black-eyed peas or lentils or peas)

Dessert Right After Lunch (Prioritize Fruits):

- 1 small unit/1 small slice (80g) of any fruit of your preference

  OR

- 2 small squares (20g) of chocolate (occasionally)

  OR

- 1 small unit (20g) of peanut candy (occasionally)

  OR

- 1 tablespoon (20g) of dulce de leche/guava paste (occasionally)

(Note: Prioritize fruits)

<u>AFTERNOON SNACK – 3:30 PM:</u>

Example 1:

Beverages:

- 1 medium cup (150ml) of coffee or unsweetened tea (if necessary, sweeten with a few drops of sweetener). Avoid drinking coffee after 3 PM.

Cereals (Carbohydrate Sources):

- 1 medium slice (25g) of whole wheat bread

  OR

- 1 small unit (25g) of French bread without the crumb

  OR

- 1 small unit (35g) of tapioca/couscous (2.5 level tablespoons)

  OR

- 2 units (20g) of whole grain or multigrain toast

  OR

- 4 units (20g) of whole grain rice cracker

Good Fat Source:

- 1 tablespoon (20g) of light ricotta cream (or light cream cheese or mashed avocado with salt and herbs)

Protein Source:

- 3 medium slices (60g) of light Minas cheese (or ricotta)

  OR

- 2 large units (120g) of eggs (boiled, poached, scrambled, omelet)

  OR

- 3 tablespoons (50g) of shredded chicken with 1 tablespoon (10g) of light ricotta cream/light cream cheese/mashed avocado with salt and herbs.

Fruits (Source of Carbohydrates and Fibers):

- 1 medium slice (100g) of papaya, pineapple, melon, watermelon, or mango

  OR

- 1 medium unit (100g) of tangerine or orange

  OR

- 1 small unit (80g) of apple, pear, banana, or guava

  OR

- 10 units (120g) of strawberry

  OR

- 8 units (100g) of grape

  OR

- 1 unit (100g) of any seasonal fruit of your choice (within appropriate portions)

Nuts and Seeds (Good Fat Source):

- 1 teaspoon (5g) of sunflower, pumpkin, sesame, chia, flaxseed, etc.

Spices and Aromatic Herbs:

- 1 coffee spoon (3g) of cinnamon to sprinkle on fruits.
- 1 coffee spoon (3g) of turmeric, black pepper, and aromatic herbs to season protein sources.

Example 2:

Protein Source:

- 1 pot (200ml) of skimmed natural yogurt

  OR

- 1 glass (250ml) of skimmed milk

  OR

- 3 medium slices (60g) of light Minas cheese/ricotta/homemade tofu

  OR

- 2 boiled eggs (120g)

Fruits (Source of Carbohydrates and Fibers):

- 1 medium slice (100g) of papaya, pineapple, melon, watermelon, or mango

  OR

- 1 medium unit (100g) of tangerine or Orange

  OR

- 1 small unit (80g) of apple, pear, banana, or guava

  OR

- 10 units (120g) of strawberry

  OR

- 8 units (100g) of grape

  OR

- 1 unit (100g) of any seasonal fruit of your choice (within appropriate portions)

Nuts or Seeds (Good Fat Source):

- 2 tablespoons (20g) of a mix of nuts (example: peanuts, almonds, Brazil nuts, walnuts, etc.)

  OR

- 1 tablespoon (10g) of sunflower seeds, pumpkin seeds, sesame seeds, chia seeds, flax seeds, etc.

Spices and Aromatic Herbs:

- 1 coffee spoon (3g) of cinnamon to sprinkle on fruits

DINNER – 7:30 PM:

Example 1:

Vegetables (Source of Good Fats):

- 1 dessert plate full (200g) of raw and/or cooked vegetables (example: lettuce, arugula, tomato, carrot, cucumber, chayote, broccoli, zucchini, eggplant, beetroot, okra, jiló, etc.)

Good Fat Source:

- 2 tablespoons (20g) of vinaigrette sauce (example: tomato, onion, soy/canola/sunflower oil, lemon or vinegar, aromatic herbs, turmeric, pepper, and a pinch of salt)

  OR

- 1 tablespoon of canola/sunflower/soy oil + ½ lemon juice or vinegar, aromatic herbs, spices, and a pinch of salt

Protein Source:

- 1 medium fillet (120g) of cooked or grilled chicken breast (or thigh without skin and no fat)

  OR

- 1 medium fillet (120g) of cooked or baked fish (sardine, panga, hake, merluza, shark, tilapia, tuna, etc.)

  OR

- 1 medium fillet (110g) of lean beef cooked or grilled (top round, eye round, bottom round, shank, tenderloin, chuck center, and rump) - Option for twice a week

  OR

- 1 medium fillet (110g) of lean pork cooked or grilled (pork tenderloin and loin)

  OR

- 2 units (120g) of eggs (boiled, poached, scrambled, omelet) - If opting for eggs at breakfast, avoid repeating at lunch/dinner

OR

- 4 medium slices (80g) of light Minas cheese or ricotta

OR

- 4 tablespoons (100g) of sautéed textured soy protein (VEGAN OPTION)

OR

- 4 tablespoons (100g) of cooked legumes (carioca beans, black-eyed peas, chickpeas, lentils, and peas) (VEGAN OPTION)

Cereals and Tubers (Carbohydrate Source):

- 3 level tablespoons (80g) of cooked rice (or cooked cassava or cooked parsley or cooked sweet potato or cooked corn or cooked pasta)

OR

- 4 level tablespoons (100g) of cooked English potato

OR

- 5 level tablespoons (120g) of cooked kabocha squash/pumpkin

Legumes (Source of Carbohydrates, Fibers, and Proteins):

- 1 medium ladle (80g) of cooked carioca beans (or black beans or black-eyed peas or lentils or peas)

Example 2:

Healthy Sandwich:

Cereals (Carbohydrate Sources):

- 2 medium slices (50g) of whole wheat bread

OR

- 1 unit (40g) of French bread without the crumb

OR

- 1 medium disc (40g) of tapioca

OR

- 1 medium disc of Rap 10 whole wheat / whole wheat pita bread (40g)

Vegetables:

- 1 dessert plate full (200g) of vegetables (example: lettuce, arugula, tomato, carrot, cucumber, cabbage, eggplant, etc.)

Good Fat Source:

- 2 tablespoons (20g) of vinaigrette sauce (example: tomato, onion, soy/canola/sunflower oil, lemon or vinegar, aromatic herbs, turmeric, pepper, and a pinch of salt)

  OR

- 1 tablespoon of canola/sunflower/soy oil + ½ lemon juice or vinegar, aromatic herbs, spices, and a pinch of salt

  OR

- 1 tablespoon (20g) of light ricotta cream (or light cream cheese or mashed avocado with salt and herbs)

Protein Sources:

- 1 medium fillet (120g) of cooked or grilled chicken breast (or thigh without skin and no fat)

  OR

- 1 medium fillet (120g) of cooked or baked fish (sardine, panga, hake, merluza, shark, tilapia, tuna, etc.)

  OR

- 1 medium fillet (110g) of lean beef cooked or grilled (top round, eye round, bottom round, shank, tenderloin, chuck center, and rump) - Option for twice a week

  OR

- 1 medium fillet (110g) of lean pork cooked or grilled (pork tenderloin and loin)

  OR

- 2 units (120g) of eggs (boiled, poached, scrambled, omelet) - If opting for eggs at breakfast, avoid repeating at lunch/dinner

  OR

- 4 medium slices (80g) of light Minas cheese or ricotta

  OR

- 4 tablespoons (100g) of sautéed textured soy protein (VEGAN OPTION)

EVENING SNACK 9:30 PM:

- 200ml of natural tea (chamomile, lemon balm, fennel, passion fruit, cinnamon, strawberry, apple, etc.) without sugar. If necessary, sweeten with a few drops of sweetener
